# Synthesis and Biological Evaluation of Herceptin-Conjugated Liposomes Loaded with Lipocalin-2 siRNA for the Treatment of Inflammatory Breast Cancer

**DOI:** 10.3390/ph18071053

**Published:** 2025-07-17

**Authors:** Marienid Flores-Colón, Mariela Rivera-Serrano, Esther A. Peterson-Peguero, Pablo E. Vivas-Rivera, Fatima Valiyeva, Pablo E. Vivas-Mejía

**Affiliations:** 1 Department of Biochemistry, University of Puerto Rico, Medical Sciences Campus, San Juan 00936, Puerto Rico; marienid.flores@upr.edu; 2 Comprehensive Cancer Center, University of Puerto Rico, San Juan 00936, Puerto Rico; mariela.rivera20@upr.edu (M.R.-S.); fvaliyeva@cccupr.org (F.V.); 3 Department of Biology, University of Puerto Rico, Río Piedras Campus, San Juan 00921, Puerto Rico; esther.peterson@upr.edu (E.A.P.-P.); pablo.vivas1@upr.edu (P.E.V.-R.)

**Keywords:** inflammatory breast cancer, lipocalin-2, liposomes, siRNA, tumor emboli

## Abstract

**Background:** Inflammatory breast cancer (IBC) is a rare and aggressive subtype of breast cancer that accounts for 1–5% of BC patients and regularly affects women under 40 years of age. Approximately 50% of IBC cases are HER2+ and can be treated with the monoclonal antibody-based therapy Herceptin (trastuzumab). However, resistance to Herceptin develops within a year, and effective second-line targeted therapies are currently unavailable for IBC patients. Lipocalin-2 (LCN2) is a promising therapeutic target for IBC due to its role in promoting tumor invasiveness, angiogenesis, and the inflammatory tumor microenvironment characteristic of IBC. **Objective:** We developed Herceptin-conjugated liposomes loaded with LCN2-targeted small-interference RNA (siRNA) for HER2+ IBCs. **Methods:** We synthesized DSPE-PEG(2000)-maleimide-Herceptin in a three-step process and formulated the liposomes together with DOPC, PEG(2000)-PE, cholesterol, and siRNA. **Results:** Dynamic light scattering confirmed the liposome size distribution, which was 66.7 nm for the Herceptin-conjugated liposome versus 43.0 nm in a non-functionalized liposome. Here, we report efficient internalization of this formulation into HER2+ IBC cells, reducing LCN2 levels by 30% and disrupting tumor emboli formation. RNA sequencing revealed 139 genes that were differentially expressed upon LCN2 knockdown, with 25 canonical pathways identified through Ingenuity Pathway Analysis. **Conclusions**: These findings suggest that LCN2-targeted siRNA within Herceptin-targeted liposomes represents a promising therapeutic strategy for IBC.

## 1. Introduction

Inflammatory breast cancer (IBC) is a rare and highly aggressive form of breast cancer (BC) that accounts for 1 to 5% of all BC cases [1,2]. Although uncommon, IBC is responsible for 8% to 10% of BC deaths [3]. The 5-year survival rate of patients with IBC is 40% to 50%, roughly half the rate for breast cancer overall [4]. As there is no specific molecular signature to identify IBC, it is often mistaken for mastitis or bacterial infections [5]. Diagnosis relies only on clinical features, including erythema and edema of the skin, peau d’orange, and increased temperature of the breast [3,6]. Additionally, the presence of tumor cell clusters, known as tumor emboli, the absence of a palpable mass in two-thirds of IBC cases, and its rapid onset make IBC highly aggressive [5,7]. Because IBC has reached these lymph vessels and has caused changes in the skin, by the time IBC is properly diagnosed, it has often metastasized, regularly to the brain, bones, and/or lungs [1,7,8,9]. Currently, aggressive treatments, including anthracycline/taxane-based chemotherapy, targeted drug therapies, surgeries (including mastectomy), and radiotherapy, are given to IBC patients due to the rapid onset of disease [7,10]. However, the poor survival rates among patients with IBC highlight a need for targeted therapies for the disease [7].

Lipocalin-2 (LCN2) is a protein involved in iron homeostasis, immune responses, siderophore transport, and epithelial cell differentiation [11]. Overexpression of LCN2 has been associated with increased cancer cell motility, epithelial-to-mesenchymal transition (EMT), proliferation, angiogenesis, invasion, and metastasis in aggressive cancers, including pancreatic, esophageal, and breast cancer [12,13]. It has been previously reported that high levels of LCN2 are found in IBC cells and patient tumors and that high LCN2 expression is associated with decreased prognosis and overall survival in patients with breast cancer [13]. Compared with non-IBC tumors, IBC tumors express high levels of LCN2, and a similar trend was observed in IBC cell lines compared with non-IBC cell lines [6,13]. Studies in our laboratory have demonstrated that siRNA-mediated knockdown of LCN2 can significantly inhibit cell proliferation and invasion in IBC cells and also induce cell cycle arrest and increased apoptosis [6].

Human epidermal growth factor receptor 2 (HER2) and triple-negative breast cancer (TNBC) are the most aggressive breast cancer molecular subtypes, associated with the highest frequency of recurrence and poorer outcomes [14,15]. IBCs are more commonly HER2+ or TNBC compared with non-IBCs, and interestingly, high levels of LCN2 have been associated with these breast cancer molecular subtypes [13,16]. A significant number of IBC tumors, approximately 50%, overexpress HER2 [17]. Unfortunately, since there is no specific treatment for IBC patients, current treatments are mostly similar to those for non-IBCs. For HER2+ breast cancer, the monoclonal antibody Herceptin (trastuzumab) is a primary therapeutic option, targeting HER2 and inhibiting cancer cell growth through mechanisms such as the disruption of HER2 homodimerization [18,19,20]. However, despite its initial effectiveness, a significant challenge remains, as most breast cancer patients develop Herceptin resistance within a year after beginning treatment [21].

Given the urgent need for novel and more effective targeted therapies for IBC patients, we developed a dual-targeted approach. We prepared and characterized a Herceptin-conjugated liposome loaded with LCN2-targeted small-interference RNA. This innovative formulation represents an advance by simultaneously targeting two critical pathways: HER2 signaling via Herceptin and LCN2-mediated aggressiveness via RNA interference using siRNA. We demonstrated that this dual-targeted strategy effectively reduced cell proliferation and tumor emboli formation in IBC cells. Furthermore, to understand the underlying molecular mechanisms of LCN2 silencing, we performed RNA sequencing in siRNA-mediated LCN2 knockdown IBC cells. By identifying the downstream signaling pathways and effectors regulated by LCN2, this study aims to propose novel molecular targets, such as TOP2A, CDCA7, and STAT1, that could lead to innovative therapeutic strategies for treating HER2+IBC and potentially overcome the current treatment limitations, such as Herceptin resistance.

## 2. Results

### 2.1. Synthesis of DSPE-PEG(2000)-Maleimide-Herceptin Conjugate Was Achieved in a Three-Step Reaction

**Scheme 1:**
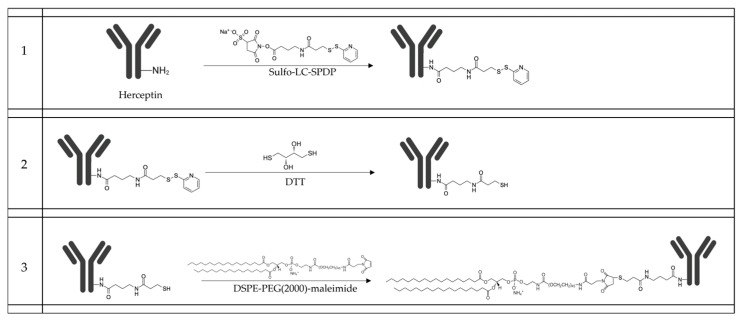
Scheme of the three consecutive reactions for the synthesis of DSPE-PEG(2000)-maleimide conjugated to Herceptin. Step 1 produces the pyridylthiol-activated Herceptin; step 2 produces the sulfhydryl-activated Herceptin; and step 3 shows the final conjugate product of DSPE-PEG(2000) maleimide-Herceptin.

**Figure 1:**
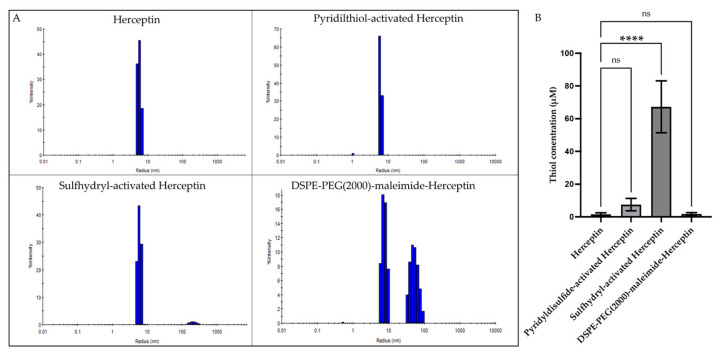
Dynamic light scattering (DLS) analysis and thiol quantification of Herceptin and Herceptin-conjugates. (**A**) DLS histograms show the size distributions of Herceptin (5.7 ± 0.4 nm), pyridylthiol-activated Herceptin (6.2 ± 0.1 nm), sulfhydryl-activated Herceptin (5.8 ± 0.3 nm), and DSPE-PEG(2000)-maleimide-Herceptin. The histography for DSPE-PEG(2000)-maleimide-Herceptin displays two particle populations at 8.4 ± 2.2 nm and 40.5 ± 12.1 nm, representing populations of different lamellarities. (**B**) Quantification of thiol concentration (μM) of Herceptin and its conjugates. The graph shows a significant increase in thiol concentration in sulfhydryl-activated Herceptin (67.2 ± 15.9 nm; **** *p <* 0.0001) compared to Herceptin alone (1.60 ± 0.4 nm) and pyridylthiol-activated Herceptin (7.4 ± 1.1 nm). Following the conjugation with DSPE-PEG(2000)-maleimide, the thiol concentration decreased to 1.69 ± 0.9 nm, confirming a successful conjugation. Statistical analysis was performed using one-way ANOVA (Dunnett’s test). **** *p* < 0.001, ns = non-significant. Data represent mean ± SD of three independent experiments (n = 3).

A schematic representation of this three-step reaction is shown in Scheme 1. First, the Herceptin antibody was initially modified by adding the sulfo-LC-SPDP group, followed by reduction of the disulfide bond to generate sulfhydryl (SH) groups in the protein (Scheme 1). The Herceptin modified with sulfhydryl was then conjugated with DSPE-PEG(2000)-maleimide via the free thiol group on the Herceptin-sulfhydryl (Scheme 1). Dynamic light scattering (DLS) was used to measure the size and polydispersity index (PDI) of each sample throughout the conjugation process. The initial size of Herceptin was 5.7 ± 0.4 nm. After the first step, the size of the pyridylthiol-activated Herceptin was 6.2 ± 0.1 nm. The following reaction with DTT produced sulfhydryl-activated Herceptin, which generated a size of 5.8 ± 0.3 nm. The final step that produces DSPE-PEG-(2000)-maleimide Herceptin generates two distinct populations with sizes of 8.4 ± 2.2 nm and 40.5 ± 12.1 nm, representing vesicles of different lamellarities. Figure 1A shows the DLS size distributions of Herceptin and each of the conjugate products. The PDI values across all the samples remained below 30%, indicating a homogeneous population. The physicochemical properties, including size distribution, PDI percentages, and percent yield of the reaction, are summarized in Table 1.

The efficiency of the conjugation was assessed by quantifying the reduced thiol groups (SH) in each step of the reactions. The thiol concentrations of Herceptin alone and pyridilthiol-activated Herceptin ranged from 1.60 μM to 7.40 μM. In contrast, sulfhydryl-activated Herceptin exhibited a significantly higher thiol concentration of 67.2 μM (*p* < 0.0001), demonstrating the abundance of free thiol groups available. Following the conjugation of DSPE-PEG(2000)-maleimide to the sulfhydryl-activated Herceptin, the thiol concentration of the final DSPE-PEG(2000)-maleimide-Herceptin conjugate was reduced to 1.69 μM. This significant decrease in the free thiol concentration is indicative of successful conjugation, as the reaction occurs through the binding of the sulfhydryl group on Herceptin to the maleimide group on the lipid. The reduction in thiol concentration is expected, as the free thiols are now covalently linked, forming a stable conjugate; thus, the thiols are no longer available or detectable. These results are depicted in Table 1 and Figure 1B. The increase in size from 5.6 ± 0.2 nm to 8.4 ± 2.2 nm, the consistent PDI values below 30%, and the decrease in thiol content from 67.2 ± 15.9 to 1.69 ± 0.90 μM confirmed the successful synthesis of the DSPE-PEG(2000)-maleimide-Herceptin conjugate.

### 2.2. The Sizes of the Herceptin-Functionalized Liposomes Loaded with siRNA Were Lower than 200 nm in Diameter

**Figure 2:**
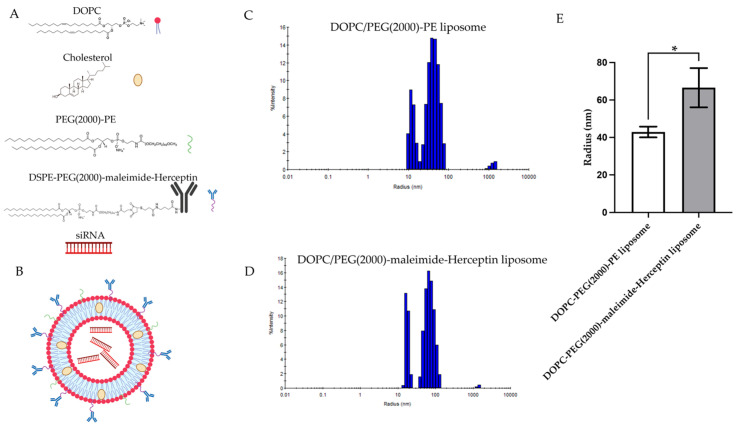
Chemical composition, assembly, and DLS size distribution of liposomal formulations. (**A**) Chemical structure of individual lipid components used in the liposome formulation, including DOPC, cholesterol, DSPE-PEG(2000)-maleimide-Herceptin conjugate, and PEG(2000)-PE. (**B**) Schematic representation of liposome assembly, demonstrating the binding of Herceptin-PEG conjugate via hydrophobic interactions and siRNA encapsulation. (**C**) Dynamic light scattering (DLS) analysis of non-functionalized liposomes showing two populations with a radius of 15.4 ± 6.8 nm and 43.0 ± 2.9 nm. (**D**) DLS analysis of Herceptin-functionalized liposomes showing increased sizes of 22.3 ± 5.7 nm and 66.7 ± 10.5 nm, consistent with successful conjugation. Each formulation was analyzed in three independent experiments (n = 3). Data are presented as mean ± Standard Deviation (SD). Polydispersity index (PDIs) ranged from 13% to 29%, confirming homogeneity of the liposomal preparations. (**E**) Statistical comparison of liposome radii showing a significant increase in size for Herceptin-functionalized liposomes compared to non-functionalized liposomes, based on unpaired two-tailed *t*-test (* *p* < 0.05).

For the synthesis of the Herceptin-conjugated liposomes, we mixed the DSPE-PEG(2000)-maleimide-Herceptin conjugate with DOPC, cholesterol, PEG(2000)-PE, and the corresponding siRNA, as described in the materials and methods section. To confirm the binding of the Herceptin conjugate to the liposomal formulation, which occurs via hydrophobic interactions, we also prepared a liposomal formulation without the Herceptin conjugate. After the lyophilized mixture was reconstituted, the liposomal formulation self-assembled (Figure 2A,B), and DLS was used to measure the diameter, polydispersity index, and charge (zeta potential) of each nanoparticle. The non-functionalized liposomes exhibited two size populations with radii of 15.4 nm and 43.0 nm. However, the Herceptin-conjugated liposomal formulation displayed increased radii of 22.3 nm and 66.7 nm in the respective populations. The observed size increase in the Herceptin-functionalized liposomes compared with the non-functionalized liposomes is indicative of a successful incorporation of Herceptin into the liposomes. In both formulations, the smaller population likely corresponds to a non-conjugated PEG(2000) micelle, whereas the larger population corresponds to the Herceptin-liposome. PDIs between 13 and 29% suggest that the populations are homogeneous. The small negative zeta potential values indicate that the liposomes are almost neutral. Table 2 summarizes the physicochemical properties of the liposomal formulations, while Figure 2C,D shows the size distributions measured using DLS, and Figure 2E shows the significant size increase (*p* = 0.0195) of the Herceptin-liposome. We have previously confirmed the size and homogeneity of similar liposome formulations by cryo-EM [22,23]. The increased particle size and homogeneity of the Herceptin-conjugated liposomal formulation confirmed the success of the synthesis.

### 2.3. Flow Cytometry Analysis Confirmed That IBC3 Cells Have Increased HER2 Receptor Levels

**Figure 3:**
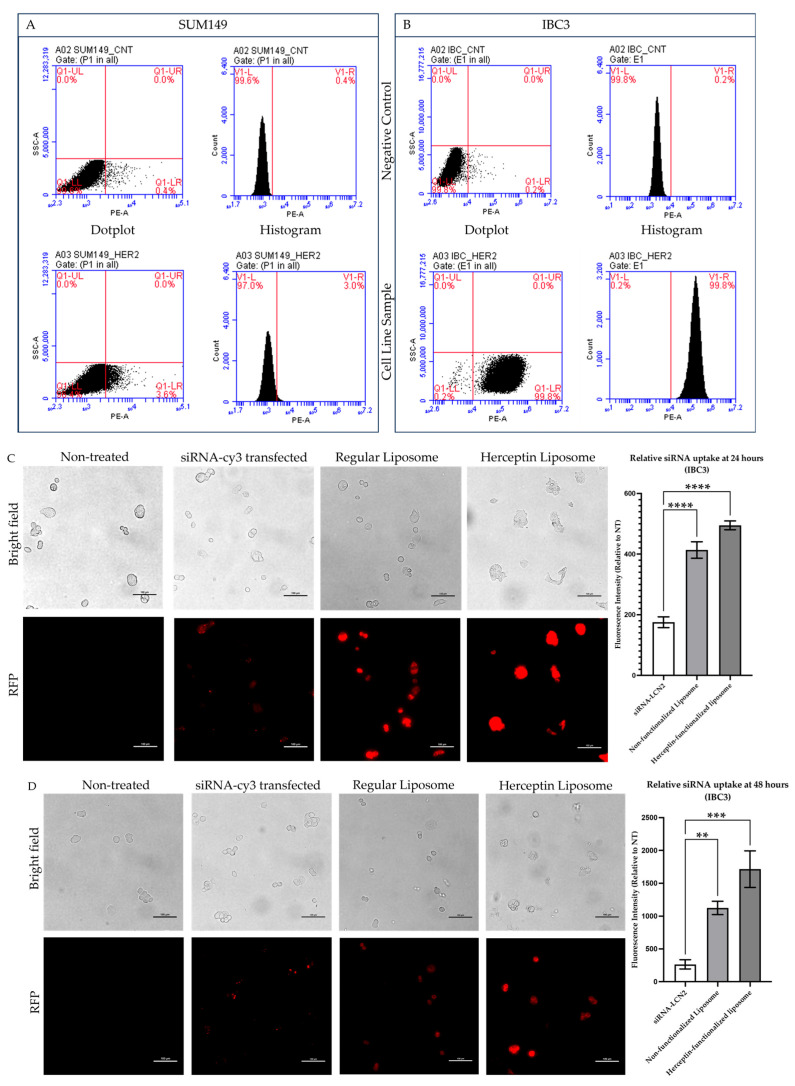

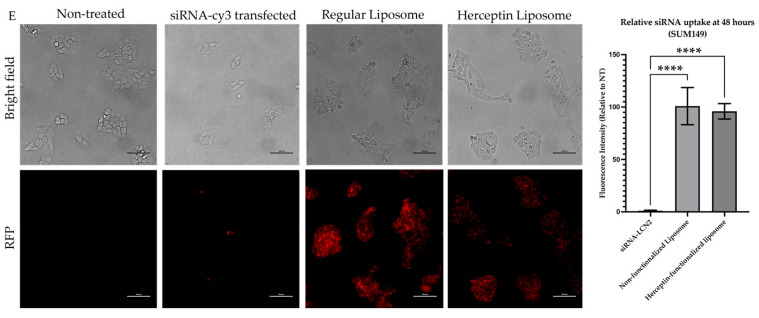
HER2 expression and siRNA/liposome internalization in IBC3 and SUM149 cells. Flow cytometry analysis was used to quantify HER2 receptor levels in (**A**) IBC3 (HER2-positive) and (**B**) SUM149 (triple-negative) cell lines. IBC3 cells exhibited high HER2 expression, with 99.8% of the population being HER2-positive, whereas only 3% of SUM149 cells were HER2-positive, validating IBC3 as an appropriate model for HER2-targeted therapies. Internalization of fluorescently labeled siRNA-Cy3 delivered via different liposomal formulations was evaluated at (**C**) 24 h and (**D**) 48 h post-treatment in IBC3 cells, and (**E**) 48 h in SUM149 cells. Each panel includes bright field (BF) and red fluorescent protein (RFP) images for four treatment conditions: untreated, siRNA-Cy3 positive control (transfected), non-functionalized liposomes, and Herceptin-functionalized liposomes. Quantification of fluorescence intensity demonstrated that Herceptin-functionalized liposomes achieved greater internalization in HER2+ IBC3 cells, with the highest uptake observed at 48 h. In contrast, non-functionalized liposomes exhibited slower and reduced uptake. Internalization was significantly lower in HER2-negative SUM149 cells, further confirming the HER2-specific targeting capabilities of the Herceptin-conjugated formulation. Experiments were done in triplicate. Images were acquired at 10× magnification using a Nikon Eclipse TS2R fluorescence microscope. Bars: three microscope fields, +/− SD, ** *p* < 0.01, *** *p* < 0.001, **** *p* < 0.0001.

To validate the use of HER2-targeted therapy in this study, we quantified the levels of HER2 expression in the IBC3 (HER2+) and SUM149 (TNBC) cell lines via flow cytometry (Figure 3A,B). IBC3 cells presented high levels of HER2 expression, with 99.8% of the cell population being HER2 positive and only 0.2% being HER2 negative (Figure 3A). On the other hand, 97% of the SUM149 were HER2-negative cells, and only 3% of the cells were HER2-positive (Figure 3B). The significant difference in HER2 expression between IBC3 and SUM149 cells validates the relevance of using IBC3 for assessing the efficacy of HER2-targeted therapies, while SUM149 can be used as an appropriate negative control.

### 2.4. Herceptin-Conjugated Liposomes Were Efficiently Internalized by HER2+ IBC Cells

To evaluate the internalization of the liposomes in HER2+ IBC cells, IBC3 and TNBC cells, and SUM149, were exposed to each formulation. The liposomes were loaded with fluorescently labeled siRNA-cyanide-3 (Cy3) negative control (NC) to monitor liposome internalization. Liposomal uptake was monitored in IBC3 cells at 2-, 8-, 24-, and 48-h post-treatment. We observed that the Herceptin-conjugated liposomes showed greater internalization as early as two hours after treatment, compared to the non-functionalized liposomes (Supplementary Figure S1A). After 8 h of treatment, both formulations were effectively internalized by HER2+ IBC cells (Supplementary Figure S1B). The highest fluorescence intensity inside cells for the Herceptin-conjugated liposomes was observed after 48 h of treatment. We observed that both Herceptin-conjugated and non-conjugated liposomes were effectively internalized into HER2+ IBC cells. The conjugated liposomes demonstrated enhanced and faster internalization, harnessing their ability to target HER2+ cells.

### 2.5. Herceptin-Conjugated Liposomes Efficiently Disrupt 3D Tumor Emboli of IBC Cells

A typical feature of IBC cells is the formation of tumor cell clusters known as tumor emboli, which block the lymph vessels and reach metastatic sites [7,24]. Thus, we assessed whether our Herceptin-conjugated liposomal formulation encapsulating siRNA against LCN2 could be effectively internalized and disrupt the formation of tumor emboli.

**Figure 4:**
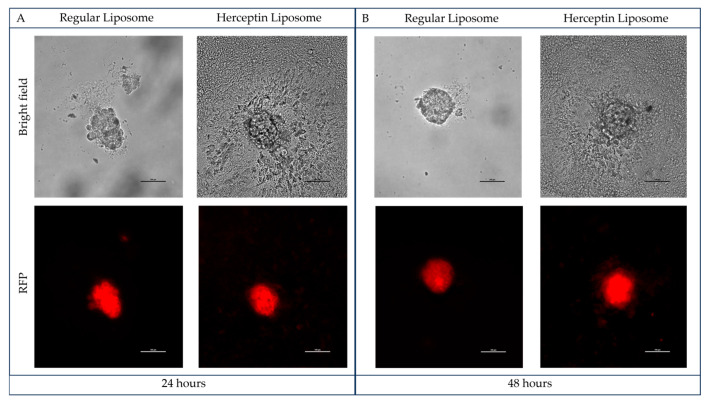

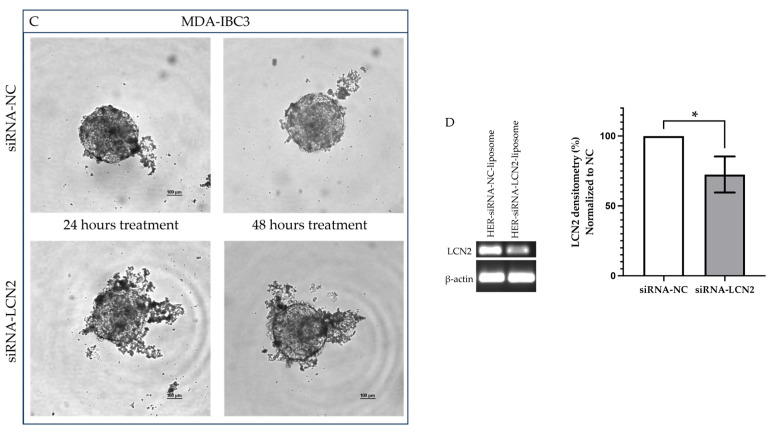
Effects of liposomes on IBC tumor emboli. Internalization and effect of non-functionalized and Herceptin-conjugated liposomes after (**A**) 24 h and (**B**) 48 h post-treatment, captured using bright field (BF) and red fluorescent protein (RFP) filters at 20× magnification. (**C**) Effect of LCN2 knockdown on IBC3 tumor emboli at 24 and 48 h. (**D**) LCN2 mRNA knockdown in HER2+ IBC3 tumor emboli treated with Herceptin-conjugated liposomes loaded with siRNA-LCN2. * *p* < 0.05. Bars: ±SD of triplicates.

We first treated IBC3 tumor emboli with Herceptin-conjugated liposomes and non-functionalized liposomes loaded with Cy3-NC siRNA for 24 and 48 h to track internalization. Fluorescence imaging indicated that both liposomal formulations were effectively internalized into the tumor emboli (Figure 4A,B). Notably, while the integrity of the tumor emboli treated with the nonfunctionalized liposomes was preserved, those treated with Herceptin-conjugated liposomes exhibited widespread degradation across the entire area of the tumor emboli structure (Figure 4A,B).

We next evaluated the impact of siRNA-NC vs. siRNA-LCN2 on IBC3 tumor emboli using a non-functionalized liposomal system. The results in Figure 4C demonstrate that the use of liposomes encapsulating an siRNA against LCN2 disrupts the integrity of the tumor emboli. Tumor emboli treated with non-functionalized-siRNA-LCN2-encapsulated liposomes presented signs of internal structural disruption, with rupture beginning within the tumor emboli (Figure 4C). In contrast, liposomes loaded with siRNA-NC preserved the structural integrity of the tumor emboli. These results suggest that LCN2 promotes tumor emboli stability.

To determine the impact of Herceptin and LCN2 targeting on the disruption of tumor emboli, we treated IC3 tumor emboli with Herceptin-conjugated liposomes loaded with siRNA-NC or siRNA-LCN2. Tumor emboli were collected 48 h post-liposomal treatment for RNA isolation and RT–PCR analysis. The PCR products were separated on an agarose gel via electrophoresis. Figure 4D shows a representative gel image in which the LCN2 and β-actin bands (loading control) are observed. Densitometric analysis of the band intensities revealed a significant reduction of approximately 30% (* *p* = 0.02) in the samples treated with Herceptin-conjugated liposomes containing siRNA-LCN2 compared with those treated with siRNA-NC (Figure 4D). These results confirmed the effective knockdown of LCN2 following treatment of tumor emboli with the Herceptin-conjugated liposomal formulation.

### 2.6. Knockdown of LCN2 in HER2+ IBC Cells Altered the Expression Levels of Transcripts Involved in Tumor Initiation and Progression

**Figure 5:**
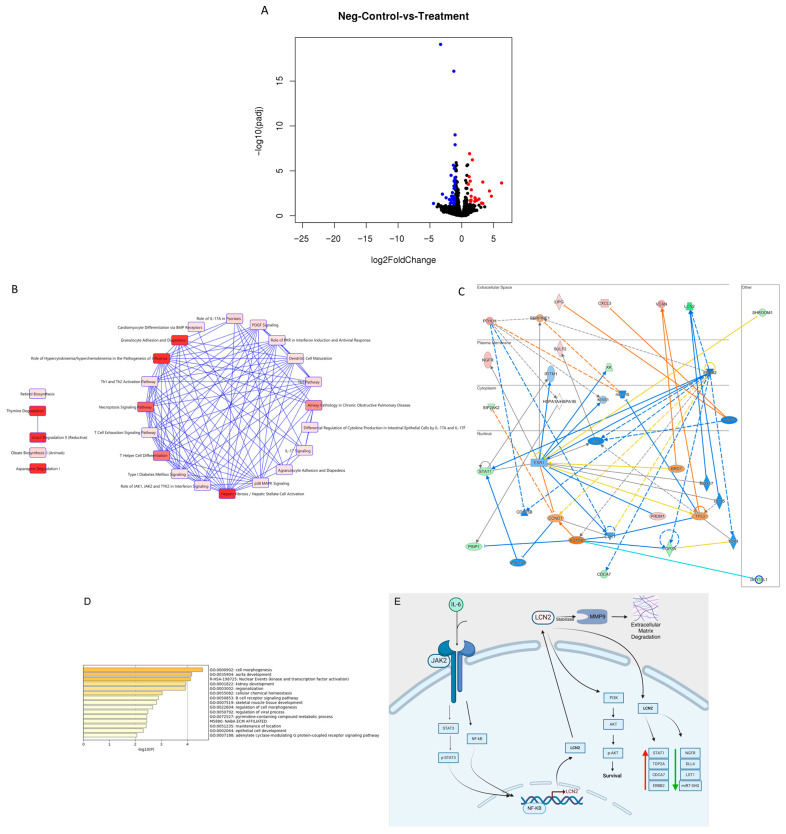
Transcriptomic and pathway analysis of dysregulated genes following LCN2 Knockdown in HER2+ MDA-IBC3 cells. (**A**) Volcano plot showing the distribution of differentially expressed genes based on log_2_FoldChange and –log_10_(adjusted *p*-value). Blue dots represent significantly downregulated genes, red dots represent significantly upregulated genes, and black dots indicate genes with no significant change (*padj* ≥ 0.05). (**B**) IPA-based identification of the top 25 canonical pathways influenced by *LCN2* knockdown. (**C**) IPA-generated network depicting functional interactions among differentially expressed genes. Nodes represent individual transcripts: red (upregulated), green (downregulated), orange (predicted activation), and blue (predicted inhibition). Solid and dashed lines denote direct and indirect interactions, respectively. (**D**) Metascape Gene Ontology and KEGG enrichment analyses display the top 15 biological processes significantly enriched among dysregulated genes (**E**), LCN2 regulatory network, and its downstream effectors. This diagram illustrates the molecular mechanisms regulating *LCN2* and its functional consequences in tumor progression. IL-6/JAK2/STAT3 signaling and NF-κB activation stimulate LCN2 transcription. PI3K/AKT signaling, influenced by LCN2, promotes cell survival. LCN2 stabilizes MMP9, promoting extracellular matrix (ECM) degradation and metastasis. RNA-seq data identified LCN2-regulated genes involved in proliferation and invasion (e.g., *STAT1*, *TOP2A*, *CDCA7*, *ERBB2*) and others with potential roles in immune modulation (e.g., *NGFR*, *DLL4*, *LST1*). Red and green arrows indicate upregulation and downregulation, respectively. Created in BioRender.

The downstream LCN2 effectors responsible for the observable biological effects of LCN2 knockdown in IBC cells have not been studied. Thus, we conducted RNA sequencing (RNA-seq) on total RNA isolated from HER2+ IBC3 cells. We generated a volcano plot to visualize the statistical significance (*p*-value) versus magnitude of change (fold change) of all genes identified by RNA-seq (Figure 5A). We identified 139 transcripts (*p* ≤ 0.05, log_2_fold change ± 1.5) that were differentially abundant (Gene Expression Omnibus database accession number GSE277628), including 82 upregulated and 57 downregulated genes, in the siRNA-LCN2 group compared with the siRNA-NC group (Supplementary Table S2). Table 3 includes the top 10 most significantly downregulated and top 10 upregulated transcripts based on their *p*-values and biological roles. Importantly, LCN2 was among the significantly downregulated genes, confirming the effectiveness of the knockdown.

To identify pathways and relationships between genes, we submitted the 139 dysregulated transcripts to the Ingenuity Pathway Analysis (IPA) software (version 23.0.1) and identified 25 canonical pathways downstream of *LCN2* (Figure 5B). These pathways are associated with key cancer processes, including proliferation, migration, survival, matrix remodeling, and angiogenesis. Specifically, 16 canonical pathways with *p*-values < 0.05 were identified (Supplementary Table S4), including granulocyte adhesion and diapedesis (*p* = 0.00534), the necroptosis signaling pathway (*p* = 0.00865), platelet-derived growth factor (PDGF; *p* = 0.0335), and p38 MAPK (*p* = 0.0443), which are some of the most relevant to this study (Table 4).

IPA also generated a gene network diagram illustrating the transcript interactions related to *LCN2* (Figure 5C). The key genes in this network included epidermal growth factor receptor-2 (*ERBB2*), which regulates cell growth and division; cell division cycle associated 7 (*CDCA7*), which contributes to metastasis and tumor invasion; and DNA topoisomerase II alpha (*TOP2A*), which is implicated in chemotherapy resistance and cell proliferation.

Further analysis of the 139 transcripts via Metascape through Gene Ontology (GO) and Kyoto Encyclopedia of Genes and Genomes (KEGG) enrichment analyses highlighted the biological processes affected by LCN2 knockdown. The top 15 significant biological processes included cell morphogenesis, aorta development, and nuclear events such as kinase and transcription factor activation (Figure 5D). Based on the most significant regulated genes of the RNA-seq and literature reports, we elaborated a signaling pathway of the LCN2 downstream effectors in IBC (Figure 5E). These comprehensive analyses revealed that LCN2 knockdown significantly altered gene expression, disrupting multiple cancer-related pathways.

## 3. Discussion

Inflammatory breast cancer (IBC) is one of the most aggressive, deadliest, and rarest forms of breast cancer, characterized by poor prognosis [9,65,66]. Despite advancements in breast cancer treatments, no specific targeted therapy has been developed for IBC. We previously reported that LCN2 is overexpressed in IBC cell lines and tumors and that high LCN2 levels are correlated with decreased overall survival in IBC patients [13]. We also showed that siRNA-mediated LCN2 knockdown in IBC cells reduced proliferation and invasion [6]. In this study, we synthesized a liposomal formulation functionalized with Herceptin and loaded it with siRNA against LCN2 to target IBC cells. This formulation was effectively internalized by HER2+ IBC cells, significantly reducing LCN2 mRNA levels and destroying the tumor emboli.

Herceptin is a monoclonal antibody commonly used as a first-line treatment in HER2+ breast cancer, including IBC, where approximately 50% of cases are HER2+ compared with 25% in non-IBC breast cancer [17]. HER2 is also overexpressed in approximately 15–20% of stomach/gastric cancers [67,68]. Due to its efficacy in targeting HER2, the US Food and Drug Administration (FDA) has approved Herceptin for use in both breast and stomach cancers [68]. HER2 activation occurs through homodimerization or heterodimerization with other HER family receptors, such as EGFR, leading to the activation of proliferative and metastatic pathways, including the MAPK and PI3K pathways [69]. However, resistance to Herceptin often develops within one to two years, leading to relapse [69,70]. This highlights the need for developing combinatory treatments that allow for lower dosages of Herceptin, delay the onset of resistance, and increase patient survival. Thus, Herceptin-conjugated liposomes are being explored to overcome acquired resistance and deliver target- and tissue-specific treatments. A study by Elamir et al. demonstrated that treating HER2+ SKBR3 BC cells with doxorubicin-loaded Herceptin-liposomes resulted in enhanced internalization, as observed through fluorescence microscopy [71]. This effect was amplified by exposing the liposomes to low-frequency ultrasound, which increased cellular uptake and decreased the viability of HER2+ SKBR3 cells [71]. Dae Hwang Shin et al. prepared a Herceptin PEGylated immunoliposome loaded with gemcitabine (GCT) [19]. Notably, this formulation has also shown antitumorigenic effects in other cancers, including ovarian, pancreatic, and breast cancers [19]. The authors demonstrated that the Herceptin-conjugated formulation was more effective than free GCT at inhibiting the proliferation of SKBR3 cells, indicating that targeted delivery significantly improved drug efficacy [19].

LCN2 is a glycoprotein that is upregulated in various types of aggressive cancers, including lung, colon, pancreatic, and breast cancer [72]. In IBC, the levels of LCN2 are elevated compared with those in non-IBC cells, regardless of the molecular subtype, making LCN2 a promising therapeutic target [6]. A recent study revealed that LCN2 also plays a role in promoting breast cancer brain metastasis [73]. LCN2 contributes to the disruption of the blood-brain barrier by activating astrocytes and microglia, increasing permeability, and promoting a pro-inflammatory microenvironment that leads to brain metastasis [73]. Within the brain parenchyma, LCN2 facilitates tumor colonization and growth by supporting neuroinflammation, modulating immune cell recruitment, and enhancing iron availability [73]. These findings suggest that LCN2 plays a role in the early stages of invasion and the establishment and progression of metastatic niches [73]. LCN2 is critical for tumor growth, epithelial-to-mesenchymal transition, angiogenesis, and cell proliferation because it forms complexes with matrix metalloproteinase-9 (MMP-9), resulting in the protection of the latter from autodegradation. Previous studies have shown that MCF-7 breast cancer cells, which naturally produce MMP-9, stably transfected to express LCN2, form tumors in xenograft models characterized by enhanced tumor growth, proliferation, and microvessel density compared to wild-type MCF-7 tumors expressing MMP-9 only [72]. Our observation of efficient internalization and targeting of LCN2 using siRNA-LCN2-loaded Herceptin-functionalized liposomes builds on these prior studies by evaluating promising innovative therapeutic avenues to inhibit the biological role of LCN2 in breast tumors.

Additional studies have focused on developing strategies for the targeted delivery of siRNA-LCN2. A liposomal formulation with an antibody targeting intercellular adhesion molecule-1 (ICAM) loaded with siRNA-LCN2 has previously shown stronger binding and internalization in triple-negative MDA-MB-231 breast cancer cells than in non-cancerous cells [74]. The formulation efficiently silenced LCN2, reduced vascular endothelial growth factor protein levels, and decreased angiogenesis in vitro. This formulation was also tested in vivo via the chick embryo chorioallantoic membrane (CAM) assay to assess angiogenesis [74]. The use of conditioned media from TNBC cells treated with PBS resulted in increased vascularization, while ICAM-functionalized liposomes significantly inhibited this effect [74]. Additionally, a separate study used a PEGylated liposomal formulation loaded with LCN2-targeted siRNA and functionalized with octreotide (OCT) for metastatic breast cancer cells, MCF-7 cells, TNBC MDA-231 cells, and normal MCF-12A cells [75]. OCT is an octapeptide analog of a growth hormone that binds to somatostatin receptors, which are highly expressed on the surface of cancer cell membranes, including those of the MCF-7 and MDA-MB-231 cell lines [75]. Compared with non-functionalized liposomes, the OCT-functionalized liposomal formulation achieved 55–60% silencing of LCN2 mRNA and reduced cell migration [75].

This report presents the synthesis and functional characterization of a novel Herceptin-targeting formulation capable of co-targeting LCN2. Liposomes in this formulation are approximately 135 nm in diameter, a size ideal for enhanced cellular uptake, as liposomes under 200 nm are known to show increased circulation and residence in the blood while enhancing tumor cell accumulation and in vivo drug release [76]. This formulation includes DOPC, PEG(2000)-PE, cholesterol, and DSPE-PEG(2000)-maleimide conjugated with Herceptin, allowing for specific targeting of HER2+ cells. The incorporation of PEG(2000)-PE enhances circulation time by preventing liposome aggregation and improving biocompatibility, whereas cholesterol increases membrane rigidity and stability, which are crucial for encapsulating siRNA [77,78]. Our results demonstrated effective liposomal internalization starting as early as 2 h post-treatment, with sustained internalization observed at 48 h. The Herceptin-conjugated liposomes disrupted tumor emboli structures in IBC, highlighting their therapeutic potential. Moreover, partial LCN2 knockdown was successfully achieved, with an approximately 30% reduction in LCN2 expression. Overall, our liposomal formulation shows potential for targeted therapy against HER2+ IBC tumors.

Our RNA-seq studies following LCN2 knockdown revealed decreased levels of key genes, including signal transducer and activator of transcription 1 (*STAT1*), cell division cycle-associated protein 7 (*CDCA7*), and topoisomerase II alpha (*TOP2A*). Direct and indirect interactions between these molecules and erb-b2 receptor tyrosine kinase 2 (*ERBB2*) were revealed via IPA network analysis. *ERBB2* is the gene that encodes HER2; our study showed its reduced expression upon *LCN2* knockdown. Similar results were obtained when HER2+ SKBR3 breast cancer cells were treated with Herceptin to block HER2, and a reduction in LCN2 expression was observed, suggesting that LCN2 may be a downstream target of the HER2 pathway [79]. These findings suggest that LCN2 knockdown alters several oncogenic pathways, providing additional potential targets for IBC treatments. Future studies should assess the effect of Herceptin-liposomes on HER2 expression in HER2+ breast cancer patients, including IBC patients. 

STAT1 is typically recognized as a tumor suppressor gene [80]. However, recent studies in breast cancer suggest that elevated STAT1 levels are associated with increased disease progression through the regulation of proinflammatory and immunosuppressive cytokines [80]. Jeon et al. studied the expression and interaction between alpha-smooth muscle actin (ACTA2) and STAT1 and reported increased levels in both HER2+ breast cancer cells and patients compared with those in HER2-negative patients [81]. The study demonstrated that overexpression of HER2 led to increases in STAT1 and ACTA2 in EGFR+ breast cancer cells, whereas the use of the STAT1 inhibitor fludarabine resulted in decreased *ACTA2* mRNA and HER2 protein levels [81]. They concluded that the JAK2/STAT1-dependent signaling pathway contributes to invasion and EMT, potentially through metastasis via the ACAT2-STAT1 axis [81]. These findings support our study, where *LCN2* knockdown reduced *STAT1* and *ERBB2* mRNA levels. Therefore, the observed reduction in both *STAT1* and *ERBB2* transcript levels upon *LCN2* knockdown may contribute to decreased EMT and metastasis, a crucial characteristic of IBCs. A recent report showed that STAT3, another member of the STAT family, plays a critical role in the resistance of IBC cell lines and IBC patients’ tumors to chemotherapy [82]. Interestingly, enhancement of STAT3 signaling leads to the upregulation of mucins (MUC1/MUC4), where MUC4 blocks the HER2 binding site, contributing to resistance to Herceptin [83].

Similarly, elevated levels of TOP2A have been observed in colon, liver, pancreatic, ovarian, and breast cancer [36,84,85]. Specifically, in breast cancer, TOP2A overexpression has been correlated with increased tumor grade and increased incidence of distant metastasis [36]. In vivo and in vitro studies in pancreatic cancer have shown that short hairpin RNA (shRNA)-mediated *TOP2A* knockdown significantly reduces cell proliferation and migration and decreases tumor size in mouse models [85]. We observed reduced expression of *TOP2A* after *LCN2* knockdown, which supports previous findings that TOP2A is a potential target in IBCs. This hypothesis needs further investigation. 

CDCA7 is a DNA-binding protein that acts as a transcriptional regulator that mediates tumor promotion and is involved in cell division [86]. Compared with normal tissues, CDCA7 is overexpressed in ovarian, colorectal, breast, and lung cancer tissues [15,87,88]. CDCA7 overexpression has been correlated with advanced clinical features, tumor development, and metastatic relapse [15]. Wang et al. reported that CDCA7 is overexpressed in lung adenocarcinoma (LUAD) patient tissues and cell lines [87]. Using siRNA-mediated knockdown of *CDCA7*, they observed decreased cell proliferation, G1 phase arrest, and increased apoptosis in the LUAD cell lines PC9 and H1299 [87]. In TNBC, elevated *CDCA7* mRNA levels are associated with increased metastasis and decreased disease-free survival [15]. ShRNA-mediated *CDCA7* knockdown significantly reduced the invasion and migration of BT549 and SUM159PT TNBC cells [15]. In our study, silencing *LCN2* led to a decrease in *CDCA7* levels in HER2+ IBC cells (RNA-seq), suggesting that *CDCA7* is a potential target in IBC.

Nerve growth factor receptor (NGFR) is a cell surface receptor involved in signal transduction pathways [58]. Studies in breast cancer, colorectal cancer, and glioblastoma suggest that NGFR possesses tumor suppressor gene capabilities; however, other studies have suggested that NGFR overexpression promotes tumor cell invasion and migration in metastatic cancers [58,59,60]. A multi-cohort microarray study revealed that only 4.5% of 245 viable invasive breast carcinoma and 38% of 37 high-grade basal-like ductal breast carcinoma samples were NGFR positive, supporting the role of NGFR as a tumor suppressor [59]. Immunohistochemistry studies using paraffin-embedded tissue samples from normal breast myoepithelial cells, benign breast lesions, and fibroadenomas (non-cancerous tumors) revealed strong reactivity of NGFR [59]. Notably, NGFR expression was significantly associated with a lack of lymph node metastasis and longer disease-free and overall survival of patients with high-grade basal-like ductal breast carcinomas [59]. Our RNA-seq data revealed that *LCN2* knockdown increased *NGFR* mRNA levels, supporting the role of NGFR as a suppressor of cell growth and proliferation in IBC cells.

One of the major pathological features of IBC is the presence of lymphovascular tumor emboli within the lymph vessels of the breast. These are responsible for the clinical features of breast swelling and erythema in more than 50% of patients [89,90,91]. These cell clusters, considered hallmarks of IBC, may constitute the primary route of cancerous cell dissemination through the blood and lymphatic vessels for distant metastasis [92]. Lehman et al. showed that growing tumor spheroids in viscous media containing PEG8000 or hyaluronic acid combined with oscillatory movements simulate the properties of tumor emboli in IBC patients [66]. They showed that while both non-IBC and IBC cell lines can form these mammospheres in Matrigel, only IBC cells can form spheroids in suspension, which they called tumor emboli [66]. Disrupting tumor emboli to avoid metastatic dissemination is a critical step toward the development of effective IBC therapies. Arora et al. demonstrated that disulfiram (an FDA-approved drug for alcohol dependence) combined with copper (DSF-Cu) disrupted 89% of TNBC SUM149 emboli relative to untreated controls [7]. A study involving HER2+ SUM190, SUM149, and multidrug-resistant rSUM149 IBC tumor emboli applied plasmonic gold nanostars for real-time imaging and photothermal ablation [91]. While incubating the cells with the nanostars alone had no significant effect on their viability, once internalized in the tumor emboli, irradiation with a near-infrared laser beam led to substantial disruption and ablation of the emboli in all three IBC cell lines, resulting in significant localized cell death [91]. Our study revealed that Herceptin-conjugated liposomal formulations were well internalized into IBC3 tumor emboli and that they had disruptive effects on emboli. While we did not interrogate specific cell death mechanisms, it is possible that *LCN2* knockdown triggered cell death pathways that compromised the internal structure of tumor emboli, leading to breakdown. Herceptin-functionalized liposomes appear to enhance external disruption through their interaction with cells, while siRNA-LCN2 seems to induce internal destabilization of the tumor emboli.

Together, our Herceptin-liposomal formulation encapsulating siRNA against lipocalin-2 (*LCN2*) offers a clinically translatable approach for HER2-positive cancers by combining targeted therapy with gene silencing. This liposomal carrier has the potential to enhance pharmacokinetics by prolonging circulation time, protecting the siRNA from degradation, and promoting tumor-specific accumulation via both the enhanced permeability and retention (EPR) effect and Herceptin-mediated targeting. This dual-action nanoparticle may surpass existing HER2-targeted therapies by not only blocking HER2 signaling but also silencing LCN2, a gene implicated in IBC progression and immune modulation. While immunogenicity remains a consideration due to potential immune responses to the liposome, siRNA, and antibody components, these risks can be mitigated through PEGylation and siRNA chemical modifications. Compared to current antibody-drug conjugates like T-DM1 or Enhertu, the formulation we are proposing here may offer improved stability and a broader therapeutic effect, making it a compelling candidate for further preclinical and clinical development [93].

## 4. Materials and Methods

### 4.1. Cell Lines and Culture Conditions

The human inflammatory breast cancer (IBC) cell lines MDA-IBC3 (HER2+) and SUM149 (triple negative, TNBC) were generously provided by Dr. Bisrat Debeb from the Department of Breast Medical Oncology at MD Anderson Cancer Center in Houston, Texas. Cells were grown in Ham’s F-12 medium (Thermo Fisher Scientific, Waltham, MA, USA) supplemented with 10% inactivated fetal bovine serum (FBS) (Thermo Fisher Scientific), 0.1% penicillin/streptomycin (Thermo Fisher Scientific), 10 µg/mL hydrocortisone (Sigma-Aldrich, St. Louis, MO, USA), and 5 µg/mL insulin from bovine pancreas (Sigma-Aldrich). All procedures were performed using cells at 70–80% cell confluency.

### 4.2. Herceptin-Conjugated Liposomal Preparation

To prepare the nanoliposome formulations, we first conjugated DSPE-PEG(2000)-maleimide to Herceptin. Briefly, the supplied lyophilized powder of Herceptin monoclonal antibody (acquired from Genentech, CA (San Francisco, CA, USA), through a material transfer agreement) was reconstituted in ultrapure water to achieve a final concentration of 21 mg/mL and stored at 4 °C until use. Three consecutive reaction steps using 1× PBS as the solvent were performed. In the first step, Herceptin (3 mg/mL) was conjugated to a sulfo-LC-SPDP crosslinker (sulfosuccinimidyl 6-(3′-[2-pyridyldithio]-propionamido) hexanoate) (Thermo Scientific cat. 21650) to produce pyridylthiol-activated Herceptin. A reduction of the disulfide bond of the pyridylthiol-activated Herceptin was subsequently performed using dithiothreitol (DTT; 23 mg/mL) as the reducing agent (2:1 ratio of pyridylthiol-activated Herceptin/DTT). Both reactions were carried out at room temperature (RT) for one hour. The thiol group (SH) of the reduced product was mixed with DSPE-PEG(2000)-maleimide (1,2-distearoyl-sn-glycero-3-phosphoethanolamine-N-[maleimide (polyethylene glycol)-2000]) (10 mg/mL resuspended in 30% ethanol; Nanosoft Polymers cat. SKU 2049-2000) combined with 28.35 μL of DSPE-PEG(2000)-maleimide with 600 μg of thiolated protein (Herceptin conjugate) and left overnight at 4 °C, as previously described [94]. Each intermediate product (Herceptin-SPDP and the thiolated Herceptin) and the final conjugate (DSPE-PEG(2000)-maleimide-Herceptin) were purified via size-exclusion chromatography (Cytiva, Marlborough, MA, USA, HiTrap Desalting 5-mL column, Sephadex G-25 medium) pre-equilibrated with 1X-PBS to remove excess unreacted reagents. The protein absorbance of all aliquots eluted from the column was measured (280 nm) via a Nanodrop 2000 Spectrophotometer (Thermo Scientific). Proteins in each aliquot were concentrated using a 50,000 molecular weight cut-off centrifugal filter (Millipore, Burlington, MA, USA). The conjugation was monitored via the Measure-IT Thiol Assay Kit (Thermo Fisher Scientific cat. M30550) to quantify free thiol groups. The size of each product was characterized using the dynamic light scattering (DLS) in a Mobius Zeta Potential Analyzer (Wyatt Technology Corporation (Santa Barbara, CA, USA)). The protein concentrations of all the Herceptin-conjugate product samples were determined using a Bio-Rad DC protein quantification kit and an xMark microplate spectrophotometer to calculate the total yield of the reaction.

Liposomes were prepared with 10 µg of siRNAs mixed with DOPC (1,2-dioleoyl-sn-glycero-3-phosphocholine) (1:10 *w*/*w* siRNA/DOPC), cholesterol (25% *w*/*w* cholesterol/DOPC), PEG(2000)-PE (1,2-distearoyl-sn-glycero-3-phosphoethanolamine-N-[methoxy(polyethylene glycol)-2000]) (Catalog: 880120 (Avanti Polar Lipids, Inc., Alabaster, AL, USA)) (10% mol/mol DOPC), and the DSPE-PEG(2000)-maleimide-Herceptin conjugate (1:1 Herceptin/DOPC). A liposomal formulation without the Herceptin conjugate was prepared as a control. The lipid mixture was diluted in 95% excess tert-butanol, frozen at −80 °C, and lyophilized. The lyophilized powder was reconstituted with 1X PBS, vortexed for 2 min, and sonicated for 15 to 20 min.

### 4.3. Liposome Internalization and LCN2 Knockdown in IBC Cells

To compare the internalization efficiency of Herceptin-functionalized and non-functionalized liposomes, we used the HER2+ IBC cell line MDA-IBC3 and the TNBC cell line SUM149. MDA-IBC3 (HER2+) and SUM149 (HER2-) cells were seeded at 4.0 × 10^4^ cells/mL in 6-well plates. Twenty-four hours later, the cells were incubated with Herceptin-functionalized and non-functionalized liposomal formulations (containing fluorescently labeled siRNA-Cyanide-3) at 37 °C. As a positive control, we transfected siRNA-Cyanide-3 using RNAiMAx (Fisher) transfection reagent at a 1:3 ratio. To determine the optimal time for liposome internalization, the cells were incubated with the formulations for 2-, 8-, 24-, or 48-h in Opti-MEM supplemented with 1% FBS. After these time periods, fluorescence images were acquired with a Nikon (Melville, NY, USA) Eclipse TS2R fluorescence microscope at 10× or 20× magnification. Bright field and red fluorescent protein (RFP) filters were used for imaging. The fluorescence intensity was analyzed using ImageJ 1.53t software (NIH, Bethesda, MD, USA).

To knockdown LCN2 levels, we used siRNA against LCN-2 targeting the sequence 5-CAUGCUAUGGUGUUCUUCA-3 (NM_005564.5; MilliporeSigma, St. Louis, MO, USA). A negative control siRNA (siRNA-NC) with the scramble sequence 5-GACCGCGAAUAGACGAACG-3 (VC30002; Millipore Sigma) was also used. MDA-IBC3 cells were seeded at 4.0 × 10^4^ cells/mL in 10 cm Petri dishes and treated the next day with the Herceptin-liposomes loaded inside with siRNA-NC or LCN2-targeted siRNA (siRNA-LCN2) at a final concentration of 300 nM. After 48 h of treatment, cell pellets were collected by centrifugation (1000 rpm for 5 min) and stored at −80 °C until use.

### 4.4. Flow Cytometry

To determine HER2 expression levels in IBC cell lines, MDA-IBC3 and SUM149 cells were harvested and collected. The cells were washed with 200 µL of ice-cold 1.5% *p*-formaldehyde/PBS for 10 min, followed by centrifugation. The resulting cell pellet was subsequently washed with 200 µL of an 80% methanol/water mixture and incubated at 4 °C for 10 min, followed by centrifugation. To minimize nonspecific binding, the cells were treated with 1% BSA/PBS at room temperature for 10 min, and the pellet was collected and resuspended in 100 µL of primary antibody solution. The cells were then incubated for 30 min with a primary antibody against human ErbB2/HER2 (R&D Systems, Minneapolis, MN, USA) (catalog: MAB1129) at 0.25 µg of antibody per million cells. An IgG2B isotype control antibody (R&D Systems cat. MAB0041) was used as a negative control. After this period, the cells were centrifuged and incubated for 10 min at room temperature with phycoerythrin anti-mouse IgG secondary antibody (R&D Systems cat. F0102B), diluted 1:200 in 1% BSA/PBS, and centrifuged again. The cells were subsequently washed twice with 1% BSA/PBS before being resuspended in 500 µL of 3% FBS/PBS. The final cell suspension was analyzed via a BD C6 Accuri flow cytometer (BD Biosciences, San Jose, CA, USA). HER2 percentages were quantified in each cell line using Accuri software (version 264.21).

### 4.5. Tumor Emboli Formation and Treatments

MDA-IBC3 cells were incubated in Hams F-12 medium (Thermo Fisher Scientific) supplemented with 10% inactivated FBS (Thermo Fisher Scientific), 0.1% penicillin/streptomycin (Thermo Fisher Scientific), 10 µg/mL hydrocortisone (Sigma-Aldrich), 5 µg/mL insulin from bovine pancreas (Sigma-Aldrich), and 2.25% PEG-8000 (Sigma-Aldrich) in a humidified atmosphere containing 5% CO_2_. IBC3 cells were plated at 1.0 × 10^4^ cells/mL in 96-well ultralow attachment, U-bottom, clear plates (EMD Millipore Corporation, Burlington, MA, USA). The plates were maintained inside the cell incubator in an OrbiShaker JR (Benchmark, Brea, CA, USA) to imitate the viscosity and shear force of the lymphatic system at a constant speed of 50 rpm. The formation of tumor emboli (also called tumor spheroids) was monitored in a Nikon Eclipse TS2R microscope using a bright field filter. After 24 h on an orbital shaker, the tumor emboli were treated with Herceptin-loaded liposomes loaded with siRNA-NC or siRNA-LCN2 at a final concentration of 300 nm. Forty-eight hours after treatment, the cells were collected and centrifuged at 1500 rpm for 5 min for RNA isolation and RT–PCR.

To evaluate the efficiency of internalization of the liposomal formulations into the tumor emboli, images of the tumor emboli were recorded with a Nikon Eclipse TS2R microscope using a bright field filter at 20× magnification. Microscopy images of the tumor emboli were also acquired at 24 and 48 h after Herceptin-liposome treatment. The area of the spheroids was measured via NIS-Elements Analysis software (version 6.10.01).

### 4.6. SiRNA Transfection, RNA Isolation, RNA Sequencing, and Data Analysis

MDA-IBC3 cells were seeded at 4.0 × 10^4^ cells/mL in Petri dishes, and a mixture of siRNAs and RNAiMax transfection reagent (at a ratio of 1:3 *v*/*v*) was added to the cells the next day. After transfection, the cells were incubated at 37 °C for 48 h, after which the cell pellets were collected for further studies.

Total RNA was isolated using the mirVana miRNA isolation kit (Fisher). Briefly, cultured cells were vortexed in 300 µL of lysis/binding solution to ensure complete lysis of the cell pellet. A volume of 1/10 of the miRNA homogenate mixture was mixed with the cell lysate and incubated on ice for 10 min. Chloroform (300 µL per sample) was added, and the mixture was vortexed and centrifuged at 10,000× *g* for 5 min to separate the aqueous and organic phases. The aqueous phase was removed, followed by the addition of 1.25 volumes of 100% ethanol. The organic phase/ethanol mixture was homogenized and passed through a filter at 10,000× *g* for 5 min. The RNA in the filter was washed three times according to the manufacturer’s instructions, and finally, the RNA was eluted from the column with 95 °C preheated nuclease-free water. DNase treatment to eliminate DNA contaminants was performed during the RNA isolation steps using a DNA-free kit (Fisher cat. AM1906). The concentration and purity of the RNA in each sample was analyzed via UV/vis spectroscopy using the NanoDrop Lite Spectrophotometer (Thermo Fisher). The RNA samples were subjected to high-throughput sequencing using an Illumina HiSeq platform (sequencing depth of 20–30 million reads per sample; raw paired-end reads per lane: 350 million; 2 × 150 base pairs configuration, single index, per lane).

### 4.7. Bioinformatic Analysis and Network Construction

Differentially abundant transcripts with *p*-values ≤ 0.05 and log_2_fold changes of ±1.5 were considered for functional network and pathway analysis using Ingenuity Pathway Analysis (IPA) software (version 23.0.1). Additionally, Metascape software (version v3.5.20250701) (https://Metascape.org/gp/index.html#/main/step1; accessed on 23 January 2023), the Gene Annotation and Analysis Resource, was used to determine the biological processes and molecular functions related to the selected transcripts. The model organism considered for each analysis was human.

## 5. Conclusions

We showed that a Herceptin-conjugated liposomal formulation loaded with siRNA-LCN2 offers a potential dual therapeutic strategy against HER2+ IBC tumor emboli, which closely mimics patient-derived IBC disease. To the best of our knowledge, this is the first study to use functionalized liposomes encapsulating siRNA for targeting *LCN2* in HER2+ IBC tumor emboli. Our findings provide insights into the molecular mechanisms involved in *LCN2* knockdown. Additionally, the data suggest potential new therapeutic targets, such as *TOP2A*, *CDCA7*, and *STAT1*, for future investigations against this aggressive form of cancer. Overall, these findings highlight the importance of LCN2 as a therapeutic target in HER2+ IBC.

## Figures and Tables

**Table 1:** Physicochemical properties of Herceptin and conjugates in the three-step reaction.

Particle	Radius (nm)	Polydispersity Index (PDI: %)	Thiol Concentration (μM)	Reaction Percent Yield (%)
Herceptin	5.6 ± 0.2	9.3± 3.0	1.6 ± 0.88	-
Pyridilthiol-activated Herceptin	6.2 ± 0.1	9.1 ± 2.8	7.4 ± 3.8	63.1 ± 8.9
Sulfhydryl-activated Herceptin	5.8 ± 0.3	14.4 ± 2.7	67.2 ± 15.9	85.5 ± 2.2
DSPE-PEG(2000)-maleimide-Herceptin	8.4 ± 2.2 40.5 ± 12.1	11.1 ± 4.5 24.2 ± 4.1	1.69 ± 0.90	81.6 ± 5.7

**Table 2:** Physicochemical properties of non-functionalized and Herceptin-conjugated liposomes. Size distribution (nm), polydispersity index (PDI, %), and zeta-potential (mV) values for non-functionalized and Herceptin-functionalized liposomal formulations. Measurements were performed using dynamic light scattering (DLS) and are presented as mean ± SD from triplicate experiments. The values highlighted in bold represent the liposome size and polydispersity index.

Particle	Radius (nm)	Polydispersity Index (PDI: %)	Zeta-Potential (mV)
DOPC-PEG(2000)-PE liposome	15.4 ± 6.8 43.0 ± 2.9	13.6 ± 4.3 28.9 ± 9.0	−0.009
DOPC-PEG(2000)-maleimide-Herceptin liposome	22.3 ± 5.7 66.7 ± 10.5	17.8 ± 6.9 27.9 ± 3.5	−0.161

**Table 3:** Top 10 upregulated and top 10 downregulated genes identified via RNA-Seq analysis.

Gene	Gene Name	log_2_fold Change	*p*-Value	Biological Role
Downregulated genes				
*LCN2*	Lipocalin-2	−3.33	5.88 × 10^−24^	Glycoproteins are associated with the transport of hydrophobic ligands, homeostasis, and epithelial cell differentiation. Associated with aggressive cancers [6]
*DPY19L1*	Dpy-19-like C-mannosyltransferase 1	−1.68	3.82 × 10^−8^	Part of the DPY19 family, predicted to enable mannosyltransferase activity. A multi-transmembrane protein known to regulate the migration of neuroblasts in *C. elegans*. Has been reported as a pro-survival gene in lung adenocarcinoma [25,26,27]
*SCD5*	Stearoyl-CoA Desaturase 5	−1.51	5.58 × 10^−5^	Involved in lipid metabolism. Overexpression is linked to increased survival in lung adenocarcinoma but shorter survival in acute myeloid carcinoma. Downregulated in breast cancer tumors [28]
*SSX2IP*	Synovial Sarcoma X breaking point 2 Interacting Protein	−1.94	0.000106	Found in normal tissues, with the highest expression in the brain. Overexpressed in various cancers, including breast and acute myeloid leukemia. Enhances tumor progression, proliferation, migration, and invasion in breast cancer [29,30]
*RDX*	Radixin	−1.60	0.000110	Membrane-cytoskeletal crosslinker in actin-rich cell surface structures. mRNA expression is higher in breast cancer tissues than in normal tissues, increasing with tumor-node-metastasis stage [31,32]
*EIF2AK2*	Eukaryotic translation initiation factor 2 alpha kinase 2	−1.64	0.000283	Regulates protein synthesis through the phosphorylation of translation initiation factor eIF2α. Expression is higher in invasive ductal carcinoma compared to normal tissue. In pancreatic cancer, high expression is related to poorer survival [33,34]
*TOP2A*	DNA topoisomerase II alpha	−1.80	0.000380	Catalytic enzyme that initiates DNA replication. In breast cancer, high levels correlate to higher tumor grade, increased incidence of distant metastasis, and shorter distant metastasis-free survival [35,36]
*STARD4*	Steroidogenic acute regulatory protein-related lipid transfer 4	−1.77	0.00118	Cholesterol transporter. Elevated levels have been associated with reduced distant metastasis-free survival in breast cancer. Has been identified as an oncogene by promoting cell proliferation and inhibiting apoptosis [37]
*SLF1*	SMC5-SMC6 complex localization factor 1	−1.61	0.00199	Has a role in DNA damage response; it is recruited together with SLF2 to DNA lesions. While it has low cancer specificity, high expression in breast cancer patients is associated with decreased survival probability [38,39,40]
*ADGRB2*	Adhesion G protein-coupled receptor B2	−1.50	0.00516	Encodes a transmembrane protein that is a brain-specific inhibitor of angiogenesis. High transcription is found in breast cancer primary tumors [41,42]
Upregulated Genes				
*KIFC3*	Kinesin family member C3	1.51	4.60 × 10^−6^	Has roles associated with centrosome cohesion, cytokinesis, vesicle transportation, and cell proliferation in mitosis. Overexpression in breast cancer is correlated to paclitaxel and docetaxel resistance; however, reports exist of decreased expression in breast cancer tumor samples [43,44,45,46]
*DLL4*	Delta-like canonical Notch ligand 4	3.30	3.40 × 10^−7^	Component of the Notch signaling pathway, involved in endothelial tip cells, related to pro-angiogenic factors. mRNA overexpression correlated to poor prognosis in various cancers, including breast and endometrial cancer, with roles in metastasis, angiogenesis, and stem cell activation [47,48,49]
*TKTL1*	Transketolase like 1	6.26	5.11 × 10^−7^	Critical for glucose metabolism and the pentose phosphate pathway. Elevated levels correlate with chemoresistance, cell proliferation, invasion, and metastasis [50]
*MIR7-3HG*	MIR7-3 host gene	4.35	6.61 × 10^−6^	Long non-coding RNA that encodes a peptide that has a protective role in pancreatic β-cells through the activation PI3K/AKT pathway [51]
*MUC22*	Mucin 22	4.65	3.39 × 10^−5^	Encodes a membrane-bound glycoprotein. mRNA expression in breast cancer showed no significant difference compared to normal controls [52]
*LST1*	Leukocyte-specific transcript 1	4.60	5.27 × 10^−5^	Encodes a small adaptor protein involved in cytoskeleton regulation. Unfavorable prognostic marker in renal and testis cancer; favorable prognostic marker in cervical cancer [53,54]
*CIART*	Circadian-associated repressor of transcription	2.08	5.87 × 10^−5^	The clock gene plays a crucial role in the synchronization of homeostatic processes. Implicated in breast cancer initiation and progression. It is overexpressed in Luminal A, grade 2, and stage 1 breast cancer [55]
*AC006372.4*	Long intergenic non-coding RNA	5.91	5.92 × 10^−5^	Annotated uncharacterized gene [56]
*IGHM*	Immunoglobulin heavy constant mu	6.62	6.23 × 10^−5^	Encodes the C region of the mu heavy chain, which defines the IgM isotype. Elevated IGHM levels are associated with increased overall survival and disease-free survival in ovarian cancer [57]
*NGFR*	Nerve growth factor receptor	2.10	7.13 × 10^−5^	Cell surface receptor with roles in cell death and survival that belongs to the tumor necrosis factor receptor superfamily. Identified as a tumor suppressor, influencing proliferation and metastasis in various breast cancer, colorectal cancer, and glioblastoma studies [58,59,60]

**Table 4:** Key canonical pathways implicated in cancer following LCN2 knockdown in HER2+ IBC cells.

Pathway	*p*-Value	Number of Genes	Genes	Biological Role
Granulocyte Adhesion and Diapedesis	0.00534	3	*CXCL3*, *NGFR*, *RDX*	An immune defense process wherein white blood cells adhere to blood vessel walls and migrate into tissues to fight infection and inflammation [61].
Necroptosis Signaling Pathway	0.00865	3	*EIF2AK2*, *NGFR*, *STAT1*	Programmed form of necrosis characterized by cell swelling and membrane rupture, typically triggered by death receptors. It functions as a defense mechanism against pathogens occurring when caspase-8 is inhibited [62]
Th1 Pathway	0.0326	2	*DLL4*, *STAT1*	A T cell differentiation pathway in which naive CD4+ T cells become Th1 cells in response to antigen and cytokine stimulation. Th1 cells produce IFN-γ and activate macrophages and cytotoxic T cells to defend against intracellular pathogens such as viruses and certain bacteria [63]
p38 MAPK	0.0443	2	*MEF2C*, *STAT1*	A stress-activated signaling pathway is triggered by inflammatory cytokines and environmental factors as UV and oxidative stress. It leads to transcription of genes involved in inflammation, cytokine production, and apoptosis [64]

## Data Availability

All data generated and analyzed during this study are included in this published article and its Supplementary Materials.

## Supplementary Materials

The following supporting information can be downloaded at: https://www.mdpi.com/article/10.3390/ph18071053/s1, Figure S1: Evaluation of siRNA-cy3 delivery using non-functionalized and Herceptin-conjugated liposomes for internalization efficiency.; Figure S2: Quantitative analysis of liposome internalization in IBC3 tumor emboli. Figure S3: Validation of RNA-Seq Results Using RT-PCR.; Table S1: Primer sequences and amplification size of the PCR experiments for RNA-seq data validation. Table S2: Differentially expressed genes following LCN2 knockdown.; Table S3: Top 25 canonical pathways identified by Ingenuity Pathway Analysis.; Table S4: Protein concentration analysis for liposomal formulations.

## References

[1] Chippa V., Barazi H. (2023). Inflammatory Breast Cancer. StatPearls[Internet].

[2] De Schepper M., Nguyen H.-L., Richard F., Rosias L., Lerebours F., Vion R., Clatot F., Berghian A., Maetens M., Leduc S. (2024). Treatment Response, Tumor Infiltrating Lymphocytes and Clinical Outcomes in Inflammatory Breast Cancer–Treated with Neoadjuvant Systemic Therapy. Cancer Res. Commun..

[3] Wang X., Semba T., Thi L., Phi H., Chainitikun S., Iwase T., Lim B., Ueno N.T. (2020). Targeting Signaling Pathways in Inflammatory Breast Cancer. Cancers.

[4] National Cancer Institute Cancer Stat Facts: Female Breast Cancer. https://seer.cancer.gov/statfacts/html/breast.html.

[5] Mamouch F., Berrada N., Aoullay Z., El Khanousi B., Errihani H. (2019). Inflammatory Breast Cancer Disease: A Literature Review. Cancer Stud..

[6] Santiago-Sánchez G.S., Noriega-Rivera R., Hernández-O’farrill E., Valiyeva F., Quiñones-Diaz B., Villodre E.S., Debeb B.G., Rosado-Albacarys A., Vivas-Mejía P.E. (2021). Targeting Lipocalin-2 in Inflammatory Breast Cancer Cells with Small Interference Rna and Small Molecule Inhibitors. Int. J. Mol. Sci..

[7] Arora J., Sauer S.J., Tarpley M., Vermeulen P., Rypens C., Van Laere S., Williams K.P., Devi G.R., Dewhirst M.W. (2017). Inflammatory Breast Cancer Tumor Emboli Express High Levels of Anti-Apoptotic Proteins: Use of a Quantitative High Content and High-Throughput 3D IBC Spheroid Assay to Identify Targeting Strategies. Oncotarget.

[8] Van Uden D.J.P., Van Maaren M.C., Strobbe L.J.A., Bult P., Van Der Hoeven J.J., Siesling S., De Wilt J.H.W., Blanken-Peeters C.F.J.M. (2019). Metastatic Behavior and Overall Survival According to Breast Cancer Subtypes in Stage IV Inflammatory Breast Cancer. Breast Cancer Res..

[9] Fernandez S.V., Robertson F.M., Pei J., Aburto-Chumpitaz L., Mu Z., Chu K., Alpaugh R.K., Huang Y., Cao Y., Ye Z. (2013). Inflammatory Breast Cancer (IBC): Clues for Targeted Therapies. Breast Cancer Res. Treat..

[10] Chainitikun S., Saleem S., Lim B., Valero V., Ueno N.T. (2021). Update on Systemic Treatment for Newly Diagnosed Inflammatory Breast Cancer. J. Adv. Res..

[11] Santiago-Sánchez G.S., Pita-Grisanti V., Quiñones-Díaz B., Gumpper K., Cruz-Monserrate Z., Vivas-Mejía P.E. (2020). Biological Functions and Therapeutic Potential of Lipocalin 2 in Cancer. Int. J. Mol. Sci..

[12] Du Z.P., Wu B.L., Xie Y.M., Zhang Y.L., Liao L.D., Zhou F., Xie J.J., Zeng F.M., Xu X.E., Fang W.K. (2015). Lipocalin 2 Promotes the Migration and Invasion of Esophageal Squamous Cell Carcinoma Cells through a Novel Positive Feedback Loop. Biochim. Biophys. Acta Mol. Cell Res..

[13] Villodre E.S., Hu X., Larson R., Finetti P., Gomez K., Balema W., Stecklein S.R., Santiago-Sanchez G., Krishnamurthy S., Song J. (2021). Lipocalin 2 Promotes Inflammatory Breast Cancer Tumorigenesis and Skin Invasion. Mol. Oncol..

[14] Mayrovitz H.N., Mayrovitz H.N. (2022). Breast Cancer.

[15] Ye L., Li F., Song Y., Yu D., Xiong Z., Li Y., Shi T., Yuan Z., Lin C., Wu X. (2018). Overexpression of CDCA7 Predicts Poor Prognosis and Induces EZH2-Mediated Progression of Triple-Negative Breast Cancer. Int. J. Cancer.

[16] What Is Inflammatory Breast Cancer?|IBC-IC|International Consortium. https://ibcic.org/what-is-ibc/.

[17] Qin R., Wang X., Fan T., Wu T., Lu C., Shao X., Yin L. (2024). Bilateral Inflammatory Recurrence of HER-2 Positive Breast Cancer: A Unique Case Report and Literature Review. Front. Oncol..

[18] Maadi H., Soheilifar M.H., Choi W., Moshtaghian A., Wang Z. (2021). Trastuzumab Mechanism of Action; 20 Years of Research to Unravel a Dilemma. Cancers.

[19] Shin D.H., Koo M.J., Kim J.S., Kim J.S. (2016). Herceptin-Conjugated Temperature-Sensitive Immunoliposomes Encapsulating Gemcitabine for Breast Cancer. Arch. Pharm. Res..

[20] Cui H., Cheng Y., Piao S.Z., Xu Y.J., Sun H.H., Cui X., Li X.Z., Zhang S.N., Piao L.Z., Jin Y.M. (2014). Correlation between HER-2/Neu(ErbB-2) Expression Level and Therapeutic Effect of Combination Treatment with HERCEPTIN and Chemotherapeutic Agents in Gastric Cancer Cell Lines. Cancer Cell Int..

[21] Hao J., Yu L., Lu H., Sakthivel T.S., Seal S., Liu B., Zhao J. (2021). Sensitization of Breast Cancer to Herceptin by Redox Active Nanoparticles. Am. J. Cancer Res..

[22] Reyes-gonzález J.M., Armaiz-peña G.N., Mangala L.S., Ivan C., Pradeep S., Echevarría-vargas I.M., Rivera- A., Sood A.K., Vivas-mejía P.E. (2016). Targeting C-MYC in Platinum-Resistant Ovarian Cancer. Mol. Cancer Ther..

[23] Reyes J., Pietri F., Vivas-Mejia P.E. (2015). Nano Based Drug Delivery.

[24] Eckhardt B.L., Ueno N.T. (2017). A Target of Potential RELAvance in Inflammatory Breast Cancer. Oncotarget.

[25] Asada K., Kobayashi K., Joutard S., Tubaki M., Takahashi S., Takasawa K., Komatsu M., Kaneko S., Sese J., Hamamoto R. (2020). Uncovering Prognosis-Related Genes and Pathways by Multi-Omics Analysis in Lung Cancer. Biomolecules.

[26] Watanabe K., Takebayashi H., Bepari A.K., Esumi S., Yanagawa Y., Watanabe K., Takebayashi H., Bepari A.K., Esumi S., Yanagawa Y. (2011). Dpy19l1, a Multi-Transmembrane Protein, Regulates the Radial Migration of Glutamatergic Neurons in the Developing Cerebral Cortex. Development.

[27] DPY19L1 Dpy-19 like C-Mannosyltransferase 1 [Homo sapiens (Human)]. https://www.ncbi.nlm.nih.gov/gene?Db=gene&Cmd=DetailsSearch&Term=23333.

[28] Zhao W., Sun L., Li X., Wang J., Zhu Y., Jia Y., Tong Z. (2021). SCD5 Expression Correlates with Prognosis and Response to Neoadjuvant Chemotherapy in Breast Cancer. Sci. Rep..

[29] Liu X., Tao M. (2023). SSX2IP as a Novel Prognosis Biomarker Plays an Important Role in the Development of Breast Cancer. Mol. Cell. Toxicol..

[30] Breslin A., Denniss F.A.K., Guinn B.A. (2007). SSX2IP: An Emerging Role in Cancer. Biochem. Biophys. Res. Commun..

[31] Hoeflich K.P., Ikura M. (2004). Radixin: Cytoskeletal Adopter and Signaling Protein. Int. J. Biochem. Cell Biol..

[32] Yuan J., Xiao C., Lu H., Yu H., Hong H., Guo C., Wu Z. (2020). MiR-200b Regulates Breast Cancer Cell Proliferation and Invasion by Targeting Radixin. Exp. Ther. Med..

[33] Du H.X., Wang H., Ma X.P., Chen H., Dai A.B., Zhu K.X. (2023). Eukaryotic Translation Initiation Factor 2α Kinase 2 in Pancreatic Cancer: An Approach towards Managing Clinical Prognosis and Molecular Immunological Characterization. Oncol. Lett..

[34] Ge L., Zhang Y., Zhao X., Wang J., Zhang Y., Wang Q., Yu H., Zhang Y., You Y. (2021). EIF2AK2 Selectively Regulates the Gene Transcription in Immune Response and Histones Associated with Systemic Lupus Erythematosus. Mol. Immunol..

[35] Zhang Y., Yang H., Wang L., Zhou H., Zhang G., Xiao Z., Xue X. (2022). TOP2A Correlates with Poor Prognosis and Affects Radioresistance of Medulloblastoma. Front. Oncol..

[36] An X., Xu F., Luo R., Zheng Q., Lu J., Yang Y., Qin T., Yuan Z., Shi Y., Jiang W. (2018). The Prognostic Significance of Topoisomerase II Alpha Protein in Early Stage Luminal Breast Cancer. BMC Cancer.

[37] Zhang M., Xiang Z., Wang F., Shan R., Li L., Chen J., Liu B.A., Huang J., Sun L.Q., Zhou W.B. (2020). STARD4 Promotes Breast Cancer Cell Malignancy. Oncol. Rep..

[38] The Human Protein Atlas. SLF1. https://www.proteinatlas.org/ENSG00000133302-SLF1/pathology/breast+cancer.

[39] Räschle M., Smeenk G., Hansen R., Temu T., Oka Y., Hein M., Nagaraj N., Long D., Walter J., Hofmann K. (2017). Proteomics Reveals Dynamic Assembly of Repair Complexes during Bypass of DNA Cross-Links. Physiol. Behav..

[40] Oravcová M., Nie M., Zilio N., Maeda S., Jami-Alahmadi Y., Lazzerini-Denchi E., Wohlschlegel J.A., Ulrich H.D., Otomo T., Boddy M.N. (2022). The Nse5/6-like SIMC1-SLF2 Complex Localizes SMC5/6 to Viral Replication Centers. eLife.

[41] ADGRB2 Adhesion G Protein-Coupled Receptor B2 [Homo sapiens (Human)]—Gene—NCBI. https://www.ncbi.nlm.nih.gov/gene/576.

[42] Shi W., Xu C., Lei P., Sun X., Song M., Guo Y., Song W., Li Y., Yu L., Zhang H. (2024). A Correlation Study of Adhesion G Protein-Coupled Receptors as Potential Therapeutic Targets for Breast Cancer. Breast Cancer Res. Treat..

[43] Tan M.-H., De S., Bebek G., Orloff M.S., Wesolowski R., Downs-Kelly E., Budd G.T., Stark G.R., Eng C. (2012). Specific Kinesin Expression Profiles Associated with Taxane Resistance in Breast Cancer. Breast Cancer Res Treat..

[44] Liao H., Zhang L., Lu S., Li W., Dong W. (2022). KIFC3 Promotes Proliferation, Migration, and Invasion in Colorectal Cancer via PI3K/AKT/MTOR Signaling Pathway. Front. Genet..

[45] Ju J.Q., Zhang H.L., Wang Y., Hu L.L., Sun S.C. (2024). Kinesin KIFC3 Is Essential for Microtubule Stability and Cytokinesis in Oocyte Meiosis. Cell Commun. Signal..

[46] Li T.F., Zeng H.J., Shan Z., Ye R.Y., Cheang T.Y., Zhang Y.J., Lu S.H., Zhang Q., Shao N., Lin Y. (2020). Overexpression of Kinesin Superfamily Members as Prognostic Biomarkers of Breast Cancer. Cancer Cell Int..

[47] Zohny S.F., Zamzami M.A., Al-Malki A.L., Trabulsi N.H. (2020). Highly Expressed DLL4 and JAG1: Their Role in Incidence of Breast Cancer Metastasis. Arch. Med. Res..

[48] Fasoulakis Z., Koutras A., Ntounis T., Pergialiotis V., Chionis A., Katrachouras A., Palios V.C., Symeonidis P., Valsamaki A., Syllaios A. (2022). The Prognostic Role and Significance of Dll4 and Toll-like Receptors in Cancer Development. Cancers.

[49] Brzozowa-Zasada M. (2021). The Role of Notch Ligand, Delta-like Ligand 4 (DLL4), in Cancer Angiogenesis—Implications for Therapy. Eur. Surg. Acta Chir. Austriaca.

[50] Hao S., Meng Q., Sun H., Li Y., Li Y., Gu L., Liu B., Zhang Y., Zhou H., Xu Z. (2022). The Role of Transketolase in Human Cancer Progression and Therapy. Biomed. Pharmacother..

[51] Mao X., Zhou J., Kong L., Zhu L., Yang D., Zhang Z. (2023). A Peptide Encoded by LncRNA MIR7-3 Host Gene (MIR7-3HG) Alleviates Dexamethasone-Induced Dysfunction in Pancreatic β-Cells through the PI3K/AKT Signaling Pathway. Biochem. Biophys. Res. Commun..

[52] Wang S., Jin J., Chen J., Lou W. (2021). MUC14-Related NcRNA-MRNA Network in Breast Cancer. Genes.

[53] The Human Protein Atlas. LST1. https://www.proteinatlas.org/ENSG00000204482-LST1/pathology.

[54] Fabisik M., Tureckova J., Pavliuchenko N., Kralova J., Balounova J., Vicikova K., Skopcova T., Spoutil F., Pokorna J., Angelisova P. (2021). Regulation of Inflammatory Response by Transmembrane Adaptor Protein LST1. Front. Immunol..

[55] Vivarelli S., Spatari G., Costa C., Giambò F., Fenga C. (2024). Computational Analyses Reveal Deregulated Clock Genes Associated with Breast Cancer Development in Night Shift Workers. Int. J. Mol. Sci..

[56] Broad Institute of MIT and Harvard Genotype-Tissue Expression (GTEx) Portal. https://www.gtexportal.org/home/gene/ENSG00000234210.1.

[57] Lu Z., Gao Y. (2021). Screening Differentially Expressed Genes between Endometriosis and Ovarian Cancer to Find New Biomarkers for Endometriosis. Ann. Med..

[58] Chen H., Huang J., Chen C., Jiang Y., Feng X., Liao Y., Yang Z. (2021). NGFR Increases the Chemosensitivity of Colorectal Cancer Cells by Enhancing the Apoptotic and Autophagic Effects of 5-Fluorouracil via the Activation of S100A9. Front. Oncol..

[59] Reis-Filho J.S., Steele D., Di Palma S., Jones R.L., Savage K., James M., Milanezi F., Schmitt F.C., Ashworth A. (2006). Distribution and Significance of Nerve Growth Factor Receptor (NGFR/P75NTR) in Normal, Benign and Malignant Breast Tissue. Mod. Pathol..

[60] Lee H., Shin C.H., Kim H.R., Choi K.H., Kim H.H. (2017). MicroRNA-296-5p Promotes Invasiveness through Downregulation of Nerve Growth Factor Receptor and Caspase-8. Mol. Cells.

[61] Granulocyte Adhesion and Diapedesis. https://geneglobe.qiagen.com/us/knowledge/pathways/granulocyte-adhesion-and-diapedesis.

[62] Dhuriya Y.K., Sharma D. (2018). Necroptosis: A Regulated Inflammatory Mode of Cell Death. J. Neuroinflamm..

[63] QIAGEN Th1 Pathway|GeneGlobe. https://geneglobe.qiagen.com/us/knowledge/pathways/th1-pathway.

[64] P38 MAPK Signaling|GeneGlobe. https://geneglobe.qiagen.com/us/knowledge/pathways/p38-mapk-signaling.

[65] Bertucci F., Finetti P., Rougemont J., Charafe-Jauffret E., Nasser V., Loriod B., Camerlo J., Tagett R., Tarpin C., Houvenaeghel G. (2004). Gene Expression Profiling for Molecular Characterization of Inflammatory Breast Cancer and Prediction of Response to Chemotherapy. Cancer Res..

[66] Lehman H.L., Dashner E.J., Lucey M., Vermeulen P., Dirix L., Van Laere S., Van Golen K.L. (2013). Modeling and Characterization of Inflammatory Breast Cancer Emboli Grown in Vitro. Int. J. Cancer.

[67] Scheck M.K., Hofheinz R.D., Lorenzen S. (2024). HER2-Positive Gastric Cancer and Antibody Treatment: State of the Art and Future Developments. Cancers.

[68] Phillips C. FDA Amends Approval of Pembrolizumab to Treat Stomach and Esophageal Junction Cancer. https://www.cancer.gov/news-events/cancer-currents-blog/2023/fda-pembrolizumab-stomach-esophageal-her2-pdl1.

[69] Cheng J., Liang M., Carvalho M.F., Tigue N., Faggioni R., Roskos L.K., Vainshtein I. (2020). Molecular Mechanism of HER2 Rapid Internalization and Redirected Trafficking Induced by Anti-HER2 Biparatopic Antibody. Antibodies.

[70] Wang Z.H., Zheng Z.Q., Jia S., Liu S.N., Xiao X.F., Chen G.Y., Liang W.Q., Lu X.F. (2022). Trastuzumab Resistance in HER2-Positive Breast Cancer: Mechanisms, Emerging Biomarkers and Targeting Agents. Front. Oncol..

[71] Elamir A., Ajith S., Al Sawaftah N., Abuwatfa W., Mukhopadhyay D., Paul V., Al-Sayah M.H., Awad N., Husseini G.A. (2021). Ultrasound-Triggered Herceptin Liposomes for Breast Cancer Therapy. Sci. Rep..

[72] Fernández C.A., Yan L., Louis G., Yang J., Kutok J.L., Moses M.A. (2005). The Matrix Metalloproteinase-9/Neutrophil Gelatinase-Associated Lipocalin Complex Plays a Role in Breast Tumor Growth and Is Present in the Urine of Breast Cancer Patients. Clin. Cancer Res..

[73] Zhao Y., Tang X., Lei T., Fu D., Zhang H. (2024). Lipocalin-2 Promotes Breast Cancer Brain Metastasis by Enhancing Tumor Invasion and Modulating Brain Microenvironment. Front. Oncol..

[74] Guo P., Yang J., Jia D., Moses M.A., Auguste D.T. (2016). ICAM-1-Targeted, Lcn2 SiRNA-Encapsulating Liposomes Are Potent Anti-Angiogenic Agents for Triple Negative Breast Cancer. Theranostics.

[75] Gote V., Pal D. (2021). Octreotide-Targeted Lcn2 Sirna Pegylated Liposomes as a Treatment for Metastatic Breast Cancer. Bioengineering.

[76] Andra V.V.S.N.L., Pammi S.V.N., Bhatraju L.V.K.P., Ruddaraju L.K. (2022). Correction to: A Comprehensive Review on Novel Liposomal Methodologies, Commercial Formulations, Clinical Trials and Patents. BioNanoScience.

[77] Li W., Huang Z., MacKay J.A., Grube S., Szoka F.C. (2005). Low-PH-Sensitive Poly(Ethylene Glycol) (PEG)-Stabilized Plasmid Nanolipoparticles: Effects of PEG Chain Length, Lipid Composition and Assembly Conditions on Gene Delivery. J. Gene Med..

[78] Hald C., Kulkarni J.A., Witzigmann D., Lind M., Petersson K., Simonsen J.B. (2022). The Role of Lipid Components in Lipid Nanoparticles for Vaccines and Gene Therapy. Adv. Drug Deliv. Rev..

[79] Leng X., Ding T., Lin H., Wang Y., Hu L., Hu J., Feig B., Zhang W., Pusztai L., Symmans W.F. (2009). Inhibition of Lipocalin 2 Impairs Breast Tumorigenesis and Metastasis. Cancer Res..

[80] Zhang M. (2013). Novel Function of STAT1 in Breast Cancer. Oncoimmunology.

[81] Jeon M., You D., Bae S.Y., Kim S.W., Nam S.J., Kim H.H., Kim S., Lee J.E. (2017). Dimerization of EGFR and HER2 Induces Breast Cancer Cell Motility through STAT1-Dependent ACTA2 Induction. Oncotarget.

[82] Stevens L.E., Peluffo G., Qiu X., Temko D., Fassl A., Li Z., Trinh A., Seehawer M., Jovanović B., Alečković M. (2023). JAK–STAT Signaling in Inflammatory Breast Cancer Enables Chemotherapy-Resistant Cell States. Cancer Res..

[83] Wang L., Wang Y., Li Y., Zhou L., Du J., Wang J., Liu S.H., Cao Y., Li Y., Yang W. (2024). Resistance Mechanisms and Prospects of Trastuzumab. Front. Oncol..

[84] Zhang K., Zheng X., Sun Y., Feng X., Wu X., Liu W., Gao C., Yan Y., Tian W., Wang Y. (2024). TOP2A Modulates Signaling via the AKT/MTOR Pathway to Promote Ovarian Cancer Cell Proliferation. Cancer Biol. Ther..

[85] Pei Y.F., Yin X.M., Liu X. (2018). qiang TOP2A Induces Malignant Character of Pancreatic Cancer through Activating β-Catenin Signaling Pathway. Biochim. Biophys. Acta Mol. Basis Dis..

[86] Li H., Weng Y., Wang S., Wang F., Wang Y., Kong P., Zhang L., Cheng C., Cui H., Xu E. (2021). CDCA7 Facilitates Tumor Progression by Directly Regulating CCNA2 Expression in Esophageal Squamous Cell Carcinoma. Front. Oncol..

[87] Wang H., Ye L., Xing Z., Li H., Lv T., Liu H., Zhang F., Song Y. (2019). CDCA7 Promotes Lung Adenocarcinoma Proliferation via Regulating the Cell Cycle. Pathol. Res. Pract..

[88] Siman L.I., Huang J., Qin M., Zhang J., Liao C. (2020). High Expression of CDCA7 Predicts Tumor Progression and Poor Prognosis in Human Colorectal Cancer. Mol. Med. Rep..

[89] Modi A.P., Nguyen J.P.T., Wang J., Ahn J.S., Libling W.A., Klein J.M., Mazumder P., Barsky S.H. (2023). Geometric Tumor Embolic Budding Characterizes Inflammatory Breast Cancer. Breast Cancer Res. Treat..

[90] Vermeulen P.B., Van Golen K.L., Dirix L.Y. (2010). Angiogenesis, Lymphangiogenesis, Growth Pattern, and Tumor Emboli in Inflammatory Breast Cancer: A Review of the Current Knowledge. Cancer.

[91] Crawford B.M., Shammas R.L., Fales A.M., Brown D.A., Hollenbeck S.T., Vo-Dinh T., Devi G.R. (2017). Photothermal Ablation of Inflammatory Breast Cancer Tumor Emboli Using Plasmonic Gold Nanostars. Int. J. Nanomed..

[92] Dobiasova B., Mego M. (2020). Biomarkers for Inflammatory Breast Cancer: Diagnostic and Therapeutic Utility. Breast Cancer Targets Ther..

[93] Gogia P., Ashraf H., Bhasin S., Xu Y. (2023). Antibody–Drug Conjugates: A Review of Approved Drugs and Their Clinical Level of Evidence. Cancers.

[94] Deng L., Zhang Y., Ma L., Jing X., Ke X., Lian J., Zhao Q., Yan B., Zhang J., Yao J. (2013). Comparison of Anti-EGFR-Fab’ Conjugated Immunoliposomes Modified with Two Different Conjugation Linkers for SiRNA Delivery in SMMC-7721 Cells. Int. J. Nanomed..

