# Supplementary material for: Synthesis and Biological Evaluation of Herceptin-Conjugated Liposomes Loaded with Lipocalin-2 siRNA for the Treatment of Inflammatory Breast Cancer
Source: Pharmaceuticals (Basel). 2025 Jul 17;18(7):1053. doi: 10.3390/ph18071053 (PMC12299312; doi:10.3390/ph18071053)
Supplement: Supplementary file 1 [file pharmaceuticals-18-01053-s001.zip › pharmaceuticals-3676602-supplementary.pdf]

pharmaceuticals

Article: Supplementary Data

Synthesis and Biological Evaluation of Herceptin-Conjugated Liposomes Loaded with Lipocalin-2 siRNA for the Treatment of Inflammatory Breast Cancer

Marienid Flores-Colón [1,2], Mariela Rivera-Serrano [2,3,†], Esther A. Peterson-Peguero [3], Pablo E. Vivas-Rivera [3,‡], Fatima Valiyeva [2] and Pablo E. Vivas-Mejía [1,2,*]

[1] Department of Biochemistry, University of Puerto Rico, Medical Sciences Campus, San Juan 00936, Puerto Rico; marienid.flores@upr.edu
[2] Comprehensive Cancer Center, University of Puerto Rico, San Juan 00936, Puerto Rico; mariela.rivera20@upr.edu (M.R.-S.); fvaliyeva@cccupr.org (F.V.)
[3] Department of Biology, University of Puerto Rico, Río Piedras Campus, San Juan 00921, Puerto Rico; esther.peterson@upr.edu (E.A.P.-P.); pablo.vivas1@upr.edu (P.E.V.-R.)
* Correspondence: pablo.vivas@upr.edu; Tel.: +17-(87)-7728300
† Current address: Cold-Spring Harbor Laboratory, Cold Spring Harbor, NY 11724, USA.
‡ Current address: Molecular Biology, Colgate University, Hamilton, NY 13346, USA.

Academic Editor: Dimitris Tsiourvas

Received: 17 May 2025
Revised: 11 July 2025
Accepted: 14 July 2025
Published: date

Citation: Flores-Colón, M.; Rivera-Serrano, M.; Peterson-Peguero, E.A.; Vivas-Rivera, P.E.; Valiyeva, F.; Vivas-Mejía, P.E. Synthesis and Biological Evaluation of Herceptin-Conjugated Liposomes Loaded with Lipocalin-2 siRNA for the Treatment of Inflammatory Breast Cancer. *Pharmaceuticals* **2025**, *18*, x. https://doi.org/10.3390/xxxxx

Copyright: © 2025 by the authors. Submitted for possible open-access publication under the terms and conditions of the Creative Commons Attribution (CC BY) license (https://creativecommons.org/licenses/by/4.0/).

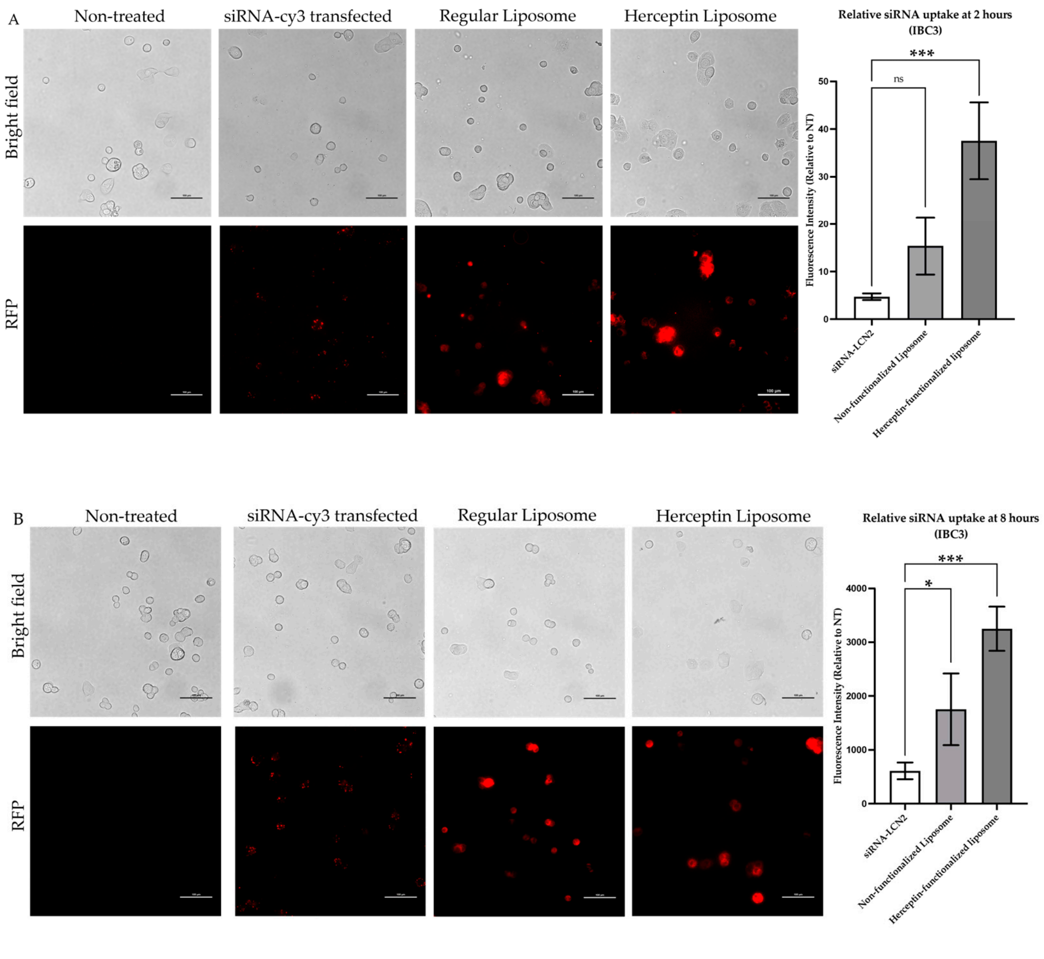

Supplementary Figure S1. Evaluation of siRNA-cy3 delivery using non-functionalized and Herceptin-conjugated liposomes for internalization efficiency. (A) 2 hours; (B) 8 hours. Each panel (A and B) includes bright field (BF) and red fluorescent protein (RFP) for non-treated cells, positive control (siRNA-cy3 transfected), non-functionalized liposome, and Herceptin-conjugated liposome. Images were captured at 10X magnification in bright field using a Nikon Eclipse TS2R fluorescent microscope. Bars: three microscope fields +/-SD. *$^{**}p<0.01$, $^{***}p<0.001$*, ns: non-statistical significance.

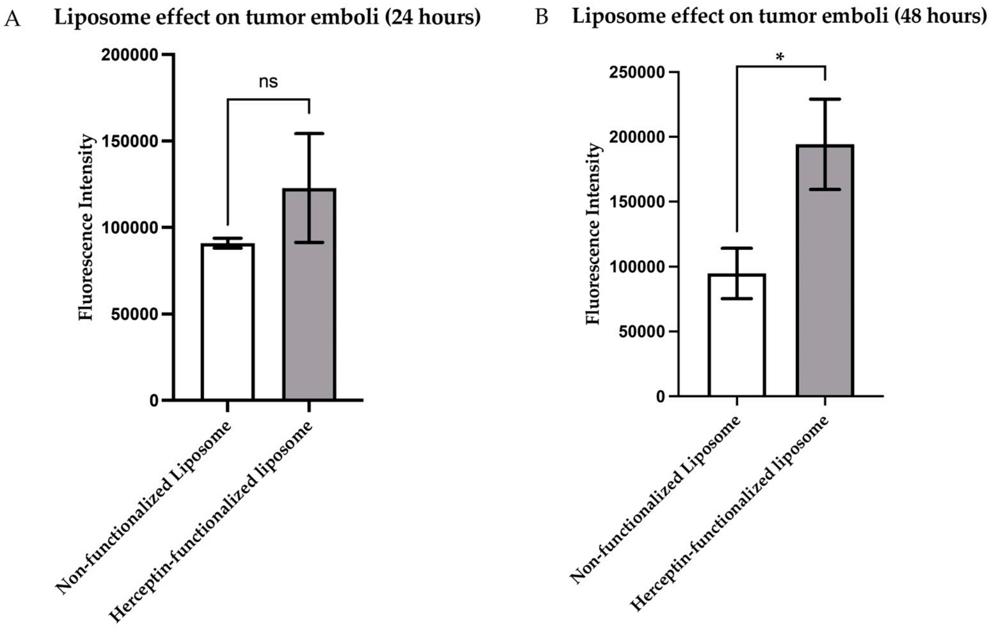

Supplementary Figure S2: Quantitative analysis of liposome internalization in IBC3 tumor emboli. Tumor emboli of IBC3 were treated with Cy3-labeled Herceptin-conjugated or non-conjugated liposomes. Fluorescence intensity was quantified using ImageJ software from representative images. (**A**) Quantification of fluorescence intensity at 24 hours post-treatment. No significant difference was observed between Herceptin-conjugated and non-conjugated liposomes. (**B**) Quantification of fluorescence intensity at 48 hours post-treatment. Herceptin-conjugated liposomes showed significantly higher fluorescence intensity compared to non-conjugated liposomes. Data are presented as mean ± SD from three independent experiments. Statistical significance was determined by an unpaired t-test. *$^{*}p = 0.0124$***.**

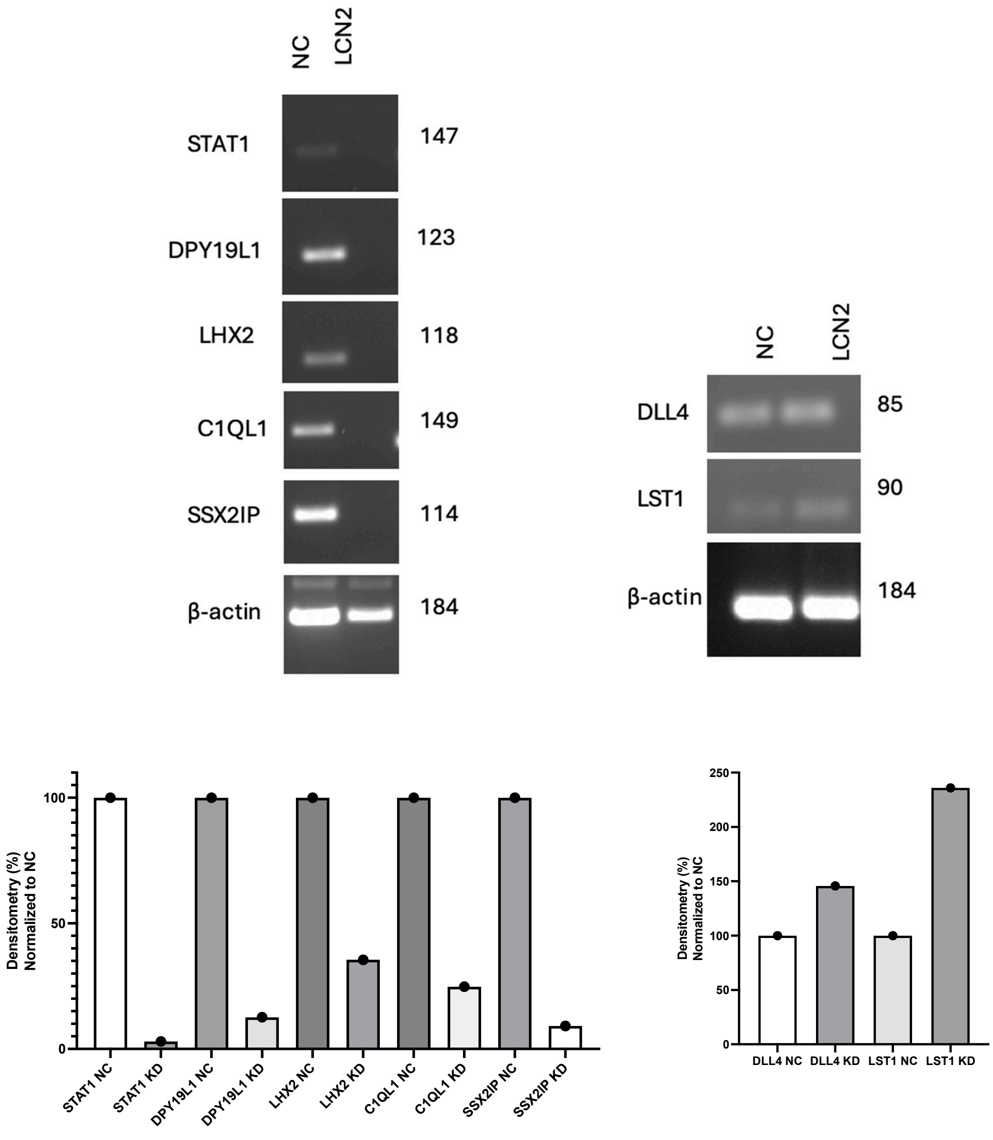

Supplementary Figure S3. Validation of RNA-Seq Results Using RT-PCR. RT-PCR analysis confirming the differential expression of selected genes following LCN2 knockdown compared to the NC. Genes analyzed include *STAT1, DPY19L1, LHX2, C1QL1, SSX2IP* (downregulated, left), *DLL4*, and *LST1* (upregulated, right). The corresponding amplicon sizes (in base pairs) are indicated on the right of each band. β-actin was used as the endogenous control to ensure RNA quality and normalization. Top: Agarose gel images. Bottom: Densitometric analysis.

Supplementary Table S1. Primer sequences and amplification size of the PCR experiments for RNA-seq data validation. For each target, two sets of primers were designed; the primer pair used for amplification is indicated in bold.

Gene Symbol	Melting Temperature (°C)	Forward Primer	Reverse Primer	Amplicon Length
STAT1	60 (multiple bands)	TGTGAAGTTGAGA-GATGTGAATGAG	AGACTGCCATTGGTG-GACTC	114
	**58**	**CTGTGAAGTTGAGA-GATGTGAATGA**	**CTTTCAATT-GCAGGTGCCGA**	**147**
DPY19L1	66	CTGGACCAC-GCTCCTGTTAG	TGCGAAAA-GCCATCTCCCTT	125
	**66**	**CCAC-GCTCCTGTTAGCTGTT**	**CAGTGCGAAAA-GCCATCTCC**	**123**
LHX2	**64**	**CCAGCTTCGGAC-CATGAAGT**	**CTGGAACCAGACCTG-GAGGA**	**118**
	64 (multiple bands)	AAAAGACGGGCCTCAC-CAAG	AAGAGGTT-GCGCCTGAACTT	76
C1QL1	**64**	**CAACATTCCCGGCAC-CTACT**	**TGCTGGCGTAGTCG-TAGTTC**	**149**
	64	CGGCAAGTTTACGTG-CAACA	GAGGTCTGCCCACAT-ACTGG	94
SSX2IP	64	TCTCACGACAAGAC-CATGAACA	GCGAGCTGCTGCTG-TAAAAG	106
	**64**	**AGCAACAGCTTTTACAG-CAGC**	**GGGACCATTCTTCTTT-GAGACG**	**114**
DLL4	-	AC-GAATGCATCCCCCACAAT	CCCATTCTTGCATGGG-GAGT	140
	**64**	**CGGGTAC-CTTCTCGCTCATC**	**ATGAGTG-CATCTGGTGGCAA**	**85**
LST1	**64**	**CAAGAGAGGCAC-CAAGGAGG**	**GTTGAG-GAAGGTGTCTGGGG**	**90**
	-	TGTTGGCTGCATCGAA-GAGT	GTGGAG-TTCCTGCTCTGAGG	93
LCN2	66°C	CAAGATCACCCTCTAC-GGGAG	ACTCAGCCGTCGATA-CACTG	141
Beta-actin	60°C	AGAGCTAC-GAGCTGCCTGAC	AGCACTGTGTTGGCG-TACAG	184

Supplementary Table S2. Differentially expressed genes following LCN2 knockdown.

Gene ID	log2FoldChange	p-value
LCN2	-3.325619929	5.88x10^{-24}
DPY19L1	-1.677101268	3.82x10^{-8}
SCD5	-1.510336119	5.58x10^{-5}
SSX2IP	-1.939925396	0.00010572
RDX	-1.604752842	0.00011011
EIF2AK2	-1.635333946	0.00028316
TOP2A	-1.79795302	0.00038
STARD4	-1.766681938	0.00118234

SLF1	-1.607218356	0.00198769
ADGRB2	-1.500208845	0.00516327
PSIP1	-1.688833242	0.00531449
AC092835.1	-2.083720846	0.00583152
RPS20P10	-4.297995971	0.0079719
XK	-1.522195391	0.0085511
MOB3B	-1.878036908	0.00916267
CDCA7	-1.806251559	0.01019132
TRIM71	-1.856058834	0.01099658
HMGB1P6	-1.619745067	0.01125238
ZC3HAV1L	-1.950736826	0.01686915
SIAE	-1.504157402	0.01757625
MEF2C	-3.492234273	0.01808442
MTCO3P22	-2.824797361	0.01816996
EGR4	-1.618612125	0.01872976
ARHGAP22	-2.7851324	0.01966845
LHX2	-2.26307241	0.02069331
MSRB3	-1.543569715	0.02259255
TMEM200C	-4.685036666	0.02309029
SLC47A1	-1.520013354	0.02341336
FBXO43	-1.54445836	0.02507311
ASRGL1	-1.706940452	0.02507313
PALD1	-1.672760621	0.02751626
RNF175	-5.703082982	0.02821103
LRP4	-1.83670315	0.02851348
AC093809.1	-2.273312546	0.02865565
DPYD	-3.35024586	0.02976042
AL022341.2	-5.65670546	0.02992527
CYP26A1	-5.00055763	0.03011288
RF00003	-2.901150645	0.0317592
WHAMMP3	-2.696413672	0.03289285
AC012531.1	-2.100103687	0.03300596
LINC01091	-1.537232503	0.03371859
EFR3B	-1.632384626	0.03456629
DUBR	-1.765600117	0.03525204
WHAMMP2	-2.850578538	0.03685005
FAM69B	-1.675831646	0.03748173
TUSC8	-2.106617396	0.03786247
SLC25A21	-1.824510154	0.03942761
C1QL1	-1.884302343	0.04112275
AL589666.2	-1.576520268	0.04160578
SHROOM1	-1.625788753	0.04285145
STAT1	-1.740153205	0.0434104
CFAP47	-1.695875943	0.04530961
NBL1	-2.352052334	0.04550038
LRRCC1	-2.035073807	0.0472864
SOX18	-2.086010365	0.04762466
AC015689.2	-1.566766095	0.04879697
NALT1	-1.55566016	0.04958985

DLL4	3.301504084	3.40×10^{-7}
TKTL1	6.255436492	5.11×10^{-7}
KIFC3	1.507425312	4.60×10^{-6}
MIR7-3HG	4.353316697	6.61×10^{-6}
MUC22	4.653262946	3.39×10^{-5}
LST1	4.603013969	5.27×10^{-5}
CIART	2.075910689	5.87×10^{-5}
AC006372.4	5.913491792	5.92×10^{-5}
IGHM	6.620654667	6.23×10^{-5}
NGFR	2.104456364	7.13×10^{-5}
KIF25-AS1	5.516115224	9.20×10^{-5}
DIO3	5.562555685	0.00024981
VCAN	3.114297741	0.00036232
ADARB2	4.033203321	0.00036717
AL122018.1	3.314282917	0.00042238
MUC16	3.134191552	0.00042244
NTNG2	2.698870533	0.00081816
SYNPO	1.786860414	0.00088429
AC010969.1	4.291678049	0.00093421
SYNE1	2.056629189	0.00109594
AL353693.1	1.879009262	0.00113454
CPNE9	3.70398309	0.00145307
EGR2	2.529293911	0.00151461
TMPRSS9	2.967006926	0.00154974
AC010319.3	3.072724925	0.00158049
SLC19A3	1.935537495	0.00159049
ARC	1.778205202	0.00160207
IGHE	3.60641913	0.00172312
GUCY2C	4.057029246	0.00207669
NPTX1	3.002470076	0.00222217
LINC02206	5.458032419	0.00230715
SLC30A2	3.212627842	0.00315969
AC135050.7	3.645370956	0.00326516
C22orf34	2.340045671	0.0044536
ATP13A5	1.919503766	0.00483242
PLK5	2.413259467	0.00488944
ARHGAP9	2.438126765	0.00491891
MXRA5	3.799950597	0.00503351
CHRM4	2.426835762	0.00620107
TSPAN2	2.872337589	0.0064404
RNU6-925P	1.776627272	0.00653352
MUC12	2.981621296	0.00706072
AC010501.1	2.98965395	0.0082733
EGOT	2.742104017	0.00858889
LINC00944	2.34230831	0.00862583
OR7E62P	2.80020749	0.00864298
REXO1L1P	2.595237101	0.00873014
LIPG	1.510577496	0.00936646
AC087203.2	3.15558916	0.0093872

AC004835.1	1.977669932	0.01052355
DUSP15	2.077185536	0.01147458
CXCL3	2.220383835	0.01157606
RFPL4A	2.448097354	0.01177184
GALR2	2.026126339	0.01226471
PTHLH	3.307688103	0.01290175
LINC00431	1.809169707	0.01406885
AC124068.2	1.520293986	0.01431701
SCRT1	4.049934398	0.01463447
PERM1	1.584870629	0.01751689
AC012435.1	1.626030725	0.02135255
PCDH19	2.453888656	0.0220988
AL023802.1	2.186874396	0.02767184
AL592430.2	1.612508473	0.03066901
SCNN1G	2.030366083	0.03408504
IRGM	2.228729905	0.03408641
DISP3	1.821124113	0.03610093
ELFN2	1.749376568	0.0364965
C5orf60	2.036281757	0.03753007
ATP1B2	1.51348679	0.0378011
AC007728.2	1.782934411	0.03814257
CNGA1	1.662726918	0.03978004
ELAVL2	1.75828572	0.04223544
SULF2	1.53911134	0.0426094
C17orf98	1.559624601	0.04288533
AL391422.3	1.64918375	0.04306636
PIK3IP1-AS1	1.745993848	0.0431614
AC137630.1	1.509876791	0.04359244
PRDM1	1.909479272	0.04382191
AC116609.1	1.603025706	0.04521083
NPHS1	1.634774694	0.04594912
GASAL1	1.508785849	0.04655633
AC139493.2	2.113739901	0.04766722

Supplementary Table S3. Top 25 canonical pathways identified by Ingenuity Pathway Analysis.

Canonical Pathways	p-value	Genes
Role of Hypercytokinemia/hyperchemokinemia in the Pathogenesis of Influenza	0.0005	3
Hepatic Fibrosis/Hepatic Stellate Cell Activation	0.0019	4
Thymine Degradation	0.0040	1
Uracil Degradation II (Reductive)	0.0040	1
Asparagine Degradation I	0.0040	1
Granulocyte Adhesion and Diapedesis	0.0053	3
Necroptosis Signaling Pathway	0.0087	3
T Helper Cell Differentiation	0.0105	2
Airway Pathology in Chronic Obstructive Pulmonary Disease	0.0145	2
Role of IL-17A Psoriasis	0.0316	1
Oleate Biosynthesis I (Animals)	0.0316	1

Th1 Pathway	0.0326	1
PDGF Signaling	0.0335	2
Differential Regulation of Cytokine Production in Intestinal Epithelial Cells by IL-17A and IL-17F	0.0393	1
Type I Diabetes Mellitus Signaling	0.0397	2
p38 MAPK Signaling	0.0443	2
Role of PKR in Interferon Induction and Antiviral Response	0.0563	2
Th1 and Th2 Activation Pathway	0.0573	2
Retinol Biosynthesis	0.0584	1
IL-17 Signaling	0.0626	2
Agranulocyte Adhesion and Diapedesis	0.0648	2
Role of JAK1, JAK2 and TYK2 in Interferon Signaling	0.0659	1
Dendritic Cell Maturation	0.0659	2
T cell Exhaustion Signaling Pathway	0.0703	2
Cardiomyocyte Differentiation via BMP Receptors	0.0734	1

Supplementary Table S4. Protein concentration analysis for liposomal formulations. Protein concentrations were measured to assess the presence of Herceptin in the liposomal formulation. The Herceptin-conjugated liposomes show a significantly higher protein concentration compared to the non-conjugated liposomes, confirming the presence of Herceptin on the surface of the conjugated liposomes. Data are presented as mean ± SD from three independent experiments.

**Particle**	**Protein concentration (μg/μL)**
Herceptin-conjugated liposome	0.118 ± 0.023
Non-conjugated liposome	0.006 ± 0.010
